# Determinants of the Efficacy of Natural Selection on Coding and Noncoding Variability in Two Passerine Species

**DOI:** 10.1093/gbe/evx213

**Published:** 2017-10-17

**Authors:** Pádraic Corcoran, Toni I Gossmann, Henry J Barton, The Great Tit HapMap Consortium, Jon Slate, Kai Zeng

**Affiliations:** * Department of Animal and Plant Sciences, University of Sheffield, South Yorkshire, United Kingdom

**Keywords:** passerine birds, effective population size, GC-biased gene conversion, adaptive evolution, purifying selection, distribution of fitness effects

## Abstract

Population genetic theory predicts that selection should be more effective when the effective population size (*N_e_*) is larger, and that the efficacy of selection should correlate positively with recombination rate. Here, we analyzed the genomes of ten great tits and ten zebra finches. Nucleotide diversity at 4-fold degenerate sites indicates that zebra finches have a 2.83-fold larger *N_e_*. We obtained clear evidence that purifying selection is more effective in zebra finches. The proportion of substitutions at 0-fold degenerate sites fixed by positive selection (*α*) is high in both species (great tit 48%; zebra finch 64%) and is significantly higher in zebra finches. When *α* was estimated on GC-conservative changes (i.e., between A and T and between G and C), the estimates reduced in both species (great tit 22%; zebra finch 53%). A theoretical model presented herein suggests that failing to control for the effects of GC-biased gene conversion (gBGC) is potentially a contributor to the overestimation of *α*, and that this effect cannot be alleviated by first fitting a demographic model to neutral variants. We present the first estimates in birds for *α* in the untranslated regions, and found evidence for substantial adaptive changes. Finally, although purifying selection is stronger in high-recombination regions, we obtained mixed evidence for *α* increasing with recombination rate, especially after accounting for gBGC. These results highlight that it is important to consider the potential confounding effects of gBGC when quantifying selection and that our understanding of what determines the efficacy of selection is incomplete.

## Introduction

Understanding the relative importance of natural selection versus genetic drift in determining the process of genome evolution is an essential task in evolutionary genetics (Kimura 1983; Gillespie 1994). It is required not just for understanding evolutionary processes such as speciation, genomic conflicts and sexual selection (Barton 2010; Hendry et al. 2011; Hosken and House 2011; Rice 2013), but also for shedding light on the spread of genetic diseases (Blekhman et al. 2008) and developing more effective conservation strategies (Allendorf et al. 2010). Theory predicts that the efficacy of both positive and purifying selection is dependent on the scaled selection coefficient *N_e_s* (Kimura 1983). Thus, all else being equal, a species with a larger *N_e_* should experience more effective purging of deleterious mutations and higher rates of fixation of beneficial variants than a species with a smaller *N_e_*. However, the efficacy of selection is also dependent on the distribution of fitness effects (DFE) of new mutations, which has been studied intensively (Eyre-Walker and Keightley 2007; Keightley and Eyre-Walker 2007; Kousathanas and Keightley 2013; Galtier 2016; Tataru et al. 2016). There is evidence that the DFE varies both across species and between different regions of the genome of a species (e.g., untranslated regions (UTRs) vs. coding regions) (Martin and Lenormand 2006; Eyre-Walker and Keightley 2007; Halligan et al. 2013; Williamson et al. 2014; Connallon and Clark 2015). Consequently, correctly determining both *N_e_* and the DFE is fundamental to understanding how natural selection has shaped genomic diversity and divergence (Keightley and Eyre-Walker 2010).

A widely used approach for quantifying the role of natural selection at the molecular level, known as DFE-*α*, uses both segregating polymorphisms and patterns of substitutions between species (Eyre-Walker et al. 2006; Keightley and Eyre-Walker 2007; Eyre-Walker and Keightley 2009; Tataru et al. 2016). This approach begins by using polymorphism data, as summarized by the site-frequency spectrum (SFS), to estimate the DFE of new deleterious mutations arising at putatively selected sites (e.g., nonsynonymous positions in protein coding regions). The estimated DFE is then used to predict the expected level of divergence at these sites between the focal species and an outgroup. Positive selection is inferred if the observed level of divergence is significantly higher than the expected, and the proportion of selected substitutions driven by positive selection, *α*, can be estimated. However, past demographic events (e.g., changes in population size and population structure) could distort the SFS and thus bias estimates of the DFE (e.g., Eyre-Walker 2002). Fortunately, simulation studies have shown that this issue can be dealt with effectively by using polymorphism data from putatively neutral sites as a control (Eyre-Walker et al. 2006; Keightley and Eyre-Walker 2007; Eyre-Walker and Keightley 2009; Messer and Petrov 2013).

Although DFE and *α* have been estimated for a wide array of organisms including humans (Boyko et al. 2008; Connallon and Clark 2015), *Drosophila* (Sella et al. 2009), fungi (Elyashiv et al. 2010; Stukenbrock et al. 2011), rodents (Halligan et al. 2013) and plants (Gossmann et al. 2010; Williamson et al. 2014), little is known about these two quantities in birds, despite the recent availability of genomes from 48 species of birds (Zhang et al. 2014). Estimating these parameters in birds is compelling for several reasons. First, birds possess a range of *N_e_* values (Ellegren 2013; Nadachowska-Brzyska et al. 2015) and several extensively studied species such as zebra finch (*Taeniopygia guttata*) (Balakrishnan and Edwards 2009; Singhal et al. 2015) have quite large *N_e_* (comparable to *Drosophila*). Thus, it is of interest to examine how these differences affect genome evolution across birds. More importantly, as discussed below, birds possess several distinctive features relating to the recombination landscape in their genome, but we still have an incomplete understanding of how they modulate evolutionary changes in birds.

Interpretation of previous estimates of DFE and *α* from studies of the chicken genome is complicated by the domestication process (e.g., artificial selection and breeding can heavily distort the SFS), small sample size and low sequencing coverage (Axelsson and Ellegren 2009; Downing et al. 2009). More recently, Galtier (2016) obtained estimates for three birds, two species of penguins and the blue tit, as part of a larger study (see further discussion below). However, the data were acquired via transcriptome sequencing, analysis of which may be complicated by difficulties in variant calling (due to, e.g., undetected paralogs [Gayral et al. 2013; Lopez-Maestre et al. 2016]), overrepresentation of highly expressed genes (which may bias estimates of DFE and α towards those genes [Galtier 2016]), and the analysis of different genes in different species (which hampers between-species comparisons). Furthermore, noncoding regions such as UTRs were not examined in any of these papers, and we currently lack estimates of the DFE and *α* in these regions in birds.

Analyses based on data from multiple species have found support for a positive relationship between *N_e_* and the intensity of natural selection (Jensen and Bachtrog 2011; Strasburg et al. 2011; Gossmann et al. 2012; Phifer-Rixey et al. 2012; Galtier 2016; Wang et al. 2016; Chen et al. 2017). Recently, Galtier (2016) tested this relationship using transcriptome data from 44 species pairs. Notwithstanding the drawbacks of transcriptome data mentioned above, Galtier (2016) found that both the strength of purifying selection and α clearly increases with *N_e_*. Interestingly, there is no clear evidence to suggest that the rate of adaptive substitutions (*ω_a_*) also increases with *N_e_*, inconsistent with the theory. Instead, the increase in α with *N_e_* is probably driven by species with greater *N_e_* fixing fewer deleterious variants rather than their accumulating beneficial substitutions at a higher rate. In birds, a number of studies have also failed to find the expected negative relationship between *d_N_/d_S_* and predictors of *N_e_* (e.g., life history traits) at both mitochondrial genes (Nabholz et al. 2013) and nuclear genes (Lanfear et al. 2010; Weber et al. 2014; Figuet et al. 2016). The reason for the absence of a relationship between *N_e_* and *ω_a_* across a number of species, or between *N_e_* and *d_N_/d_S_* in birds (but see Botero-Castro et al. 2017), is unclear, and more data from other species would help to further elucidate the relationship between *N_e_* and selection.

The efficacy of natural selection may also covary with recombination rate across the genome of a species. This has been hypothesized to be brought about by a process known as Hill-Robertson interference (HRI) (Hill and Robertson 1966; Felsenstein 1974). The HRI theory predicts that regions of low recombination should experience stronger interference between selected loci, due to a greater degree of linkage between sites. This will result in a greater reduction of *N_e_* in these regions, relative to regions that experience higher rates of recombination. Thus, we expect a positive relationship between the efficacy of selection and recombination rate. Although evidence has been found in several species (e.g., *Drosophila melanogaster* [McGaugh et al. 2012; Campos et al. 2014]; *Caenorhabditis* [Cutter and Choi 2010]), no such relationship was found in humans (Bullaughey et al. 2008) and some plant species (Slotte et al. 2011; Flowers et al. 2012). Indeed, mixed evidence has been found in *Saccharomyces cerevisiae* where correlation based analyses support higher efficacy of selection in regions of high recombination (Pál et al. 2001; Connallon and Knowles 2007; Weber and Hurst 2009), whereas a multiple regression analysis failed to identify recombination rate as a predictor of divergence at nonsynonymous sites (*d_N_*) (Cutter and Moses 2011). Thus, the importance of HRI as a modulator of selection remains uncertain (Cutter and Payseur 2013).

Birds have a highly heterogeneous recombination landscape, making them an interesting test case for the HRI model. This stems from the fact that karyotype is highly conserved in birds (Griffin et al. 2007) and is composed of chromosomes of very different sizes, ranging from macrochromosomes ∼196 Mb in length (chromosome 1 in chicken) to microchromosomes smaller than 10 Mb (Ellegren 2013). Because at least one crossover per chromosome is needed for proper segregation during meiosis, this variation in size means that the per-site recombination rate is much higher on microchromosomes than macrochromosomes. For instance, in the great tit (*Parus major*) genome, the average recombination rates on chromosomes 2 (its largest chromosome ∼150 Mb in length) and 22 (a microchromosome ∼4 Mb in length) are 0.81 and 16.54 cM/Mb, respectively (van Oers et al. 2014). In contrast, the average rates for human chromosomes 2 and 22 are 1.07 and 2.10 cM/Mb, respectively (Jensen-Seaman et al. 2004). Furthermore, in the two birds we investigate here, great tits and zebra finches, there is extensive intrachromosome variation in recombination frequency on macrochromosomes, with most of the recombination concentrated around chromosome ends, contrasting with large internal sections (“deserts”) of reduced recombination (Stapley et al. 2008; Backstrom et al. 2010; van Oers et al. 2014). However, previous investigations have come to different conclusions regarding the importance of HRI as a modulator of the efficacy of selection in birds. Using divergence patterns (as summarized by *d_N_*/*d_S_* ratios), Gossmann et al. (2014) suggested that both positive and purifying selection are more effective in high-recombination regions in the great tit genome, but these authors did not have access to polymorphism data, making it hard to tease apart the relative contribution of positive and purifying selection. In a study of the flycatcher genome, Bolívar et al. (2016) did not find evidence for HRI. Thus, analysis with both polymorphism and divergence data in additional species of birds is required to resolve these conflicting findings.

A confounding factor, which has attracted less attention in previous applications of DFE-α and related methods that involve estimating *N_e_s* from polymorphism data, is GC-biased gene conversion (gBGC; Duret and Galtier 2009). This recombination-associated neutral process leads to the preferential transmission of G/C nucleotides to the descendants of GC/AT heterozygotes. This creates a selection-like force favoring G/C nucleotides (Nagylaki 1983). gBGC has been shown to be an important factor in determining variation in genomic GC content in many different organisms (Pessia et al. 2012), including birds (Webster et al. 2006; Nabholz et al. 2011; Weber et al. 2014), although there are uncertainties as to its importance in *Drosophila* (e.g., Jackson et al. 2017).

It has been shown that gBGC can increase the frequency of deleterious polymorphisms in the population (Necşulea et al. 2011) and elevate *d_N_*/*d_S_* by driving fixation of slightly deleterious alleles (Galtier et al. 2009; Ratnakumar et al. 2010). Given that the strength of gBGC is expected to be stronger in high-recombination regions (e.g., Glémin et al. 2015), it is possible that gBGC could lead to spurious correlation between recombination rate and the efficacy of selection. Accounting for the confounding effects of gBGC is particularly important when studying selection and HRI in birds (Bolívar et al. 2016). This is because the karyotype is highly conserved, the recombination rate is inherently high on microchromosomes, and the location of recombination hotspots seems to be conserved across species (Singhal et al. 2015). These factors mean that gBGC may have a particularly strong effect on genome evolution by acting on homologous genomic regions persistently over a long period (Mugal et al. 2013).

In this study, we investigate the role of natural selection on a genome-wide scale in two wild passerine species: the great tit (Laine et al. 2016) and the zebra finch (Warren et al. 2010). These two extensively studied species have large effective population sizes, although *N_e_* may be substantially different between them (Singhal et al. 2015; Laine et al. 2016), making them good systems for addressing the questions raised below. We generated a new great tit polymorphism data set consisting of ten birds sequenced to ∼44× coverage. For the zebra finch, we obtained the genomes of ten wild individuals sequenced to ∼22× coverage by Singhal et al. (2015). Using these data we sought to address the following questions. How widespread are positive and purifying selection in these two birds? Is the intensity of purifying selection and the prevalence of adaptive substitution different between coding regions and UTRs? How different is *N_e_* between the two species, and does this difference translate into both positive and purifying selection being more effective in the species with a larger *N_e_*? Is the extent of HRI an important determinant of variation in the efficacy of selection within the genomes of these two species? In all these analyses, we explicitly controlled for the confounding effects of gBGC. In addition, we analyzed a theoretical model and asked to what extent ignoring gBGC would lead to biased estimation of the distribution of fitness effects of new mutations, overestimation of the prevalence of adaptive substitution, and spurious positive relationships between the rate of adaptive evolution and recombination rate.

## Materials and Methods

### Sampling and Sequencing

We carried out whole-genome resequencing for ten male great tits across Europe. Although the level of differentiation between different great tit populations is generally very low (*F_ST_* < 0.02; Laine et al. 2016), as an extra precaution, we adopted a scattered sampling scheme by sequencing one individual per population from ten European great tit populations (supplementary table S1, Supplementary Material online), which should further reduce the effects of population structure (Wakeley 1999). Paired-end library preparation and whole-genome sequencing using the Illumina HiSeq 2500 platform were carried out at BGI Hong Kong (read length = 125 bp; insert size ≈ 475). An initial round of quality filtering of the FASTQ files containing the reads was performed by BGI, which led to the removal of adapter sequences, contamination and low-quality reads. We searched for any remaining traces of adapter contamination in the reads using FastQC v0.11.3 (Andrews 2010) and used cutadapt v1.8.1 (Martin 2011) to remove any residual adapter sequences.

We aligned the quality filtered reads to the great tit reference genome (v1.04) (Laine et al. 2016) using BWA-MEM v0.7.12-r1039 with default settings (Li 2013) and converted the alignments to BAM format using samtools v1.2 (Li et al. 2009). Following the GATK best practice recommendations (https://software.broadinstitute.org/gatk/best-practices/; last accessed October 20, 2017), we marked PCR duplicates using Picard’s MarkDuplicates (http://broadinstitute.github.io/picard/; last accessed October 20, 2017) and performed local realignment around INDELs on the BAM files of each sample using RealignerTargetCreator and IndelRealigner in GATK v3.4 (McKenna et al. 2010).

For the zebra finch, we chose a sample of ten individuals from the Fowlers Gap population in Australia (supplementary table S1, Supplementary Material online) for which whole-genome resequencing data were publicly available (Singhal et al. 2015). These data were sequenced using Illumina HiSeq 2000 with 100 bp paired-end reads. An INDEL realigned and base quality score recalibrated BAM file for each of the ten zebra finch individuals, prepared as described by Singhal et al. (2015), was downloaded from http://www.ebi.ac.uk/ena/data/view/PRJEB10586, last accessed October 20, 2017.

### SNP Calling and Filtering

We performed an initial round of variant calling in each species using the GenotypeGVCF and HaplotypeCaller tools in GATK v3.4 (All command line options used in the SNP calling and filtering pipeline can be found on https://github.com/padraicc/Corcoran_et_al_2017; last accessed October 20, 2017). The variants in this initial call set were then hard filtered according to the GATK recommendations for variants derived from DNA sequencing. This hard-filtered set of variants was used as known variants for the base quality score recalibration (BQSR) step of the GATK best practice pipeline (Van der Auwera et al. 2013). We obtained a new set of variants by repeating the variant calling procedure on these recalibrated BAM files. VCF files were generated using the GenotypeGVCF program with the option “-includeNonVariantSites” which outputs genotype calls at both the variant and nonvariant positions. To perform variant quality score recalibration (VQSR), we needed to have a set of known SNPs as a training set. To this end, we carried out variant calling using the program Freebayes v1.02 (Garrison and Marth 2012), and identified SNPs called by both GATK and Freebayes. These SNPs were further filtered by excluding SNPs with lower than 0.5× or higher than 2× the mean depth of coverage across samples and with a QAUL score <20. We used this filtered set of SNPs as our training set in the VQSR. We set a tranche level cut-off of 99% for the zebra finch sample and 99.9% for the great tit sample. Tranche cut-offs were chosen based upon visual inspection of the tranche plots produced by GATK, and were in line with the difference in coverage between the two data sets (∼22× for the zebra finch and ∼44× for the great tit). The variant and nonvariant sites that fell within repetitive regions were excluded from further analyses. Additionally, we excluded variant and nonvariant sites with lower than 0.5× or higher than 2× the mean depth of coverage across samples (Singhal et al. 2015). All analyses described below were based on autosomal sites, both variant and nonvariant, where a genotype call was made in every sample and at most two alleles were present.

### Annotation, Ortholog Detection and Alignment Pipeline for Divergence Estimates

We downloaded annotation for the great tit genome from the NCBI at ftp://ftp.ncbi.nlm.nih.gov/genomes/all/GCF/001/522/545/GCF_001522545.1_Parus_major1.0.3, last accessed October 20, 2017. This annotation file was produced for version 1.0.3 of the reference genome, which precedes the 1.0.4 version used in this study. However, the chromosomal sequences are identical between these two assembly versions. We also downloaded the NCBI annotation for the zebra finch genome from ftp://ftp.ncbi.nlm.nih.gov/genomes/all/GCF/000/151/805/GCF_000151805.1_Taeniopygia_guttata-3.2.4, last accessed October 20, 2017.

To ensure high alignment quality, we used a gene-by-gene approach to obtain divergence estimates in coding regions between the chicken, zebra finch and great tit genomes. We downloaded the refseq annotations for the three species. We focused on the longest predicted transcript for each gene. To identify one-to-one orthologs between zebra finch and chicken, we conducted a reciprocal best-hit search using blastp (Altschul et al. 1990). A great tit gene was added to a zebra finch-chicken orthologous gene pair if it hit the relevant gene in the pair in the two separate reciprocal best-hit searches against the chicken and zebra finch genomes, respectively. The resulting orthologous gene triplets were further filtered for cases in which the HUGO Gene Nomenclature Committee (HGNC; http://www.genenames.org; last accessed October 20, 2017) identifier was inconsistent between species. The orthologous gene triplets were then aligned using MUSCLE (Edgar 2004). Regions with poor alignment quality were identified using ZORRO (Wu et al. 2012) and were removed, resulting in the removal of 17% of sites. We then estimated *d_N_* and *d_S_* using PAML (one-ratio model) (Yang 2007) and excluded triplets with extreme substitution rate estimates (*d_N_*> 2 and *d_S_*> 5). This filtering resulted in 8,638 triplet gene alignments. For each species, we excluded genes that were not located on an autosome, contained premature stop codons or lacked any called sites in the filtered VCF. These filters resulted in a set of 7,799 genes analyzed in the zebra finch and 8,095 genes for the great tit.

We used a whole genome alignment approach to obtain divergence estimates for the UTRs. We downloaded the reference genomes for chicken (v5.0; Hillier et al. 2004), zebra finch (v3.2.4; Warren et al. 2010) and great tit (v1.0.4; Laine et al. 2016). First we created pairwise alignments with the zebra finch as a reference using LASTZ (Harris 2007), following the procedures described in previous analyses of avian genomes (Jarvis et al. 2014; Zhang et al. 2014). This was followed by chaining and netting using axtChain and chainNet, respectively (Kent et al. 2003). Finally, single coverage was ensured for the reference genome using single_cov2.v11 from the MULTIZ package and the pairwise alignments were aligned with MULTIZ (Blanchette et al. 2004). Coordinates for 5′ UTRs and 3′ UTRs in the zebra finch and great tit genomes were obtained from their respective annotation databases, and were analyzed together in each species. Only the UTRs of genes in the orthologous gene set described above were analyzed. This resulted in UTR alignments for 4,524 genes.

### Calculating Polymorphism Based Summary Statistics

For each species, we used the annotation files to identify 0-fold degenerate (hereafter 0-fold) and 4-fold degenerate (hereafter 4-fold) sites in the VCF files for the genes in the orthologous gene set. We calculated nucleotide diversity (π; Tajima 1983), Watterson’s θ (Watterson 1975) and Tajima’s *D* (Tajima 1989) separately for 0-fold and 4-fold sites. To control for the effects of gBGC, we assigned A/T or G/C polymorphisms (either 0-fold or 4-fold) as weak-to-weak (WW) or strong-to-strong (SS), respectively, and recalculated π, θ and Tajima’s *D* on weak-to-weak and strong-to-strong (WWSS) polymorphisms. In addition to controlling for gBGC, using WWSS variants also removes C → T and G → A transition mutations at methylated CpG sites, which occur at high rates due to rapid deamination. In protein-coding regions, if transitions arising at CpG sites are under stronger selective constraints than those arising in non-CpG contexts, as has been reported in humans (Schmidt et al. 2008), differences between results obtained from all sites and those obtained from WWSS sites may be in part caused by CpG hypermutability. The potential confounding effects between CpG hypermutability and gBGC are discussed further in the Discussion. The summary statistics were calculated similarly on the UTR data. We obtained 95% confidence intervals (CIs) for the statistics by calculating them on each of 10,000 bootstrap replicate data sets we generated by randomly sampling genes with replacement.

### Divergence Estimates in Protein Coding Genes and UTRs

Our analysis requires estimates of the following quantities along each of the two evolutionary lineages leading to the great tit and the zebra finch: 1) substitution rates at 0-fold and 4-fold sites (referred to as *d*_0_ and *d*_4_, respectively), and 2) the actual numbers of 0-fold and 4-fold substitutions. There is evidence that base composition is not at equilibrium in many avian lineages (Nabholz et al. 2011; Mugal et al. 2013; Weber et al. 2014), and when this is the case, using an equilibrium model such as those implemented in CODEML in PAML may lead to biased estimates (Matsumoto et al. 2015). Therefore, we used an alternative approach. We concatenated the alignments in the orthologous gene set and extracted 0-fold and 4-fold sites. We used the BASEML program from the PAML package v4.9 (Yang 2007) to estimate substitution rates separately from the 0-fold and 4-fold sites alignments. We ran BASEML first with the equilibrium GTR substitution model and then with the nonequilibrium GTR-NH_b_ model (Matsumoto et al. 2015). Likelihood ratio tests suggest that the GTR-NH_b_ model is a better fit to the data in all cases. To obtain estimates of branch specific substitution rates and the number of substitutions, we used a method developed by Matsumoto et al. (2015), which is based on reconstructing the ancestral sequence for the common ancestor of zebra finch and great tit, using posterior predictions of ancestral states generated by the GTR-NH_b_ model. Uncertainties in these predictions were taken into account by weighting the four possible nucleotides at each site in the ancestral genome by their posterior probabilities, as detailed in Matsumoto et al. (2015) (referred to as the AWP method therein). This reconstruction method has been shown to be much more reliable than maximum parsimony and maximum likelihood methods that assume base composition equilibrium (Matsumoto et al. 2015). We obtained 95% CIs by analyzing 100 bootstrap replicate data sets generated by randomly sampling genes with replacement. We used the same approach to calculate lineage-specific substitution rates for the UTRs.

### Estimating the Distribution of Fitness Effects (DFE) and the Prevalence of Adaptive Substitutions

We used the program DFE-α v2.15 to estimate parameters of the distribution of fitness effects (DFE) of new deleterious mutations and to quantify the amount of adaptive substitutions at 0-fold sites and the UTRs for each species (Keightley and Eyre-Walker 2007; Eyre-Walker and Keightley 2009). In these analyses, we used the folded SFS and 4-fold sites as the neutral reference. First, we fitted a demographic model to the SFS for neutral sites using maximum likelihood (ML). We chose a two-epoch demographic model that allows a single step change in population size from *N*_1_ to *N*_2_*t*_2_ generations in the past (Keightley and Eyre-Walker 2007). We performed multiple ML searches, each with a different starting point, and treated the parameter values that produced the highest log-likelihood as the ML estimates of the demographic parameters. Next, given the estimated parameters of the demographic model, we inferred the DFE by fitting a gamma distribution to the SFS for the selected sites. As above, we carried out multiple searches with different starting values for β and $\overset{-}{s}$, where β is the shape parameter of the gamma distribution and $\overset{-}{s}\;$is the mean fitness effect of deleterious mutations. The ML estimates of the DFE parameters and the observed divergence at the selected and neutral sites were then used to estimate the proportion of substitutions that have been fixed by positive selection (α) and the relative rate of adaptive substitution (*ω*_a_) (Eyre-Walker and Keightley 2009). To understand the effects of gBGC, we performed the analysis detailed above using either all variants or only WWSS changes. We obtained 95% CIs for the parameter estimates by analyzing 100 bootstrap replicate SFS and divergence data sets generated by randomly sampling genes with replacement.

### Gene Binning Methods for Studying Variation in the Efficacy of Selection within Genome

Testing for a relationship between predictors of local *N_e_* (e.g., recombination rate) and the efficacy of natural selection within the genome of each species requires binning genes according to these predictors. Here we chose to use three bins primarily because, as shown in the Results, controlling for gBGC is essential, but using only WWSS changes resulted in a ∼8-fold reduction in the number of polymorphic sites at our disposal. Therefore, using a small number of bins should prevent genuine signals from being overwhelmed by statistical noise while allowing us to capture the major effects of the predictor of interest.

We considered two different predictors of local *N_e_*: 1) 4-fold site GC content (GC_4_), and 2) local gene density, as measured by the *M*/*C* ratio, where *M* and *C* are, respectively, the map length and the number of coding sites in the focal window. As reported in Results, GC_4_ is correlated with the recombination rate for the gene (derived from the genetic map; see below) in these two species, and is likely to reflect the long-term recombination environment the gene has been exposed to (Mugal et al. 2013; Gossmann et al. 2014; Kawakami et al. 2014). The *M*/*C* binning was intended to control for the known positive correlation between local recombination rate and gene density in birds (e.g., Backstrom et al. 2010). Under the HRI theory, local *N_e_* should relate positively to *M*/*C* (e.g., Charlesworth 2012; Cutter and Payseur 2013). To estimate the recombination rate, we first fitted a third-order polynomial curve to the genetic map position as a function of physical position for makers on each chromosome using the genetic maps available for each species (Stapley et al. 2008; van Oers et al. 2014). This approach has been reported to be less sensitive to regional variation in recombination rates than the sliding window approaches, and to be robust to errors in the physical and genetic maps (Fiston-Lavier et al. 2010). The polynomial provided a good fit to the data in both species (*R*^2^ ≥ 0.94 and ≥0.84, for all autosomes in great tit and zebra finch, respectively). The recombination rate for a given position was then estimated as the derivative of the polynomial curve at that point. To estimate an *M*/*C* for each gene, we defined a window with the center at the midpoint of the gene and the two boundaries ±500 Kb from the midpoint. The average recombination rate for the window (in cM/Mb) was taken as the average of the recombination rates estimated at the midpoint and the two boundaries (which should be appropriate given the resolution of the genetic maps). The number of coding sites in each window was calculated using the annotation information.

### gBGC Model

Assume a diploid model with constant population size *N*. The fate of WWSS mutations arising in neutral regions is unaffected by gBGC, such that their fixation probability and SFS follow those predicted by the standard neutral model. In contrast, under gBGC, W → S mutations in neutral regions behave as though they were favored by weak selection, with strength *B* = 4*Nb*, where *b* ≥ 0 is the intensity of the conversion bias (Nagylaki 1983), whereas S → W mutations in neutral regions behave as though they were disfavored with intensity -*B*. For selected mutations, it is assumed that their fitness effects follow a distribution with density function *f*(*γ*) where *γ * = 4*Ns* and *s* is the fitness difference between homozygotes for the wild type and heterozygotes, with positive (negative) values signifying beneficial (deleterious) mutations. We assume that the effects of gBGC and selection combine additively. Thus, in selected regions, the fitness effects of WWSS mutations are unaffected by gBGC, whereas the fates of W → S and S → W mutations are determined by *γ  *+ *B* and *γ* – *B*, respectively. The mutation process is modeled as follows. Let *u* be the mutation rate per site per generation between A and T nucleotides, between G and C nucleotides, and from A/T to G/C (for simplicity the transition/transversion mutational bias is not explicitly considered). But the mutation rate from G/C to A/T is *κu*, where *κ* is the mutational bias parameter, measuring the extent to which the mutation process is biased towards A/T. The GC content is denoted by *p*, and is assumed to be constant over the time period considered, which is a reasonable approximation given the very slow rate at which GC content changes.

First consider the SFS. The total number of WWSS mutations entering the population each generation is 2 *N*[*pu* + (1 – *p*)*u*] = 2*Nu* = *θ*/2, where *θ *= 4*Nu*. The number of W → S mutations is (1 – *p*)*θ*/2, and that of S → W mutations is *pκθ*/2. Considering a sample of size *n*, the SFSs for these three types of mutations in a selected region are given, respectively, by:

(1) $$\psi_{wwss}(i)=\theta \int_{\gamma }\int_{0}^{1}\tau (\gamma ,x,i)f(\gamma )dxd\gamma$$

(2) $$\psi_{ws}(i)=(1-p)\theta \int_{\gamma }\int_{0}^{1}\tau (\gamma +B,x,i)f(\gamma )dxd\gamma$$

and

(3) $$\psi_{sw}(i)=p\kappa \theta \int_{\gamma }\int_{0}^{1}\tau (\gamma -B,x,i)f(\gamma )dxd\gamma$$

where 1 ≤ *i *<* n* and

(4) $$\tau (\gamma ,x,i)=\frac{1-e^{-\gamma (1-x)}}{\left(1-e^{-\gamma })x(1-x\right)}\left(\begin{array}{c} n\\ i \end{array}\right)x^{i}{\left(1-x\right)}^{n-i}$$

Next consider the divergence process. Let *T* be the divergence time, in units of 4 *N* generations, between the ingroup and outgroup species. The divergence levels for the three types of sites are, respectively,

(5) $$K_{wwss}=T\theta \int \frac{\gamma }{1-e^{-\gamma }}f(\gamma )d\gamma$$

(6) $$K_{ws}=T(1-p)\theta \int \frac{\gamma +B}{1-e^{-(\gamma +B)}}f(\gamma )d\gamma$$

and

(7) $$K_{sw}=Tp\kappa \theta \int \frac{\gamma -B}{1-e^{-(\gamma -B)}}f(\gamma )d\gamma$$

The total divergence is given by *K *=* K_wwss_* + *K_ws_* + *K_sw_*.

We examined how ignoring gBGC may affect the estimation of the DFE, *α*, and *ω_a_*. We assumed that all new mutations arising in the selected region are deleterious and that the DFE follows a (reflected) gamma distribution, i.e., −*γ* ∼ Gamma(*β*, $-\overset{-}{\gamma }$), where *β* is the shape parameter and $\overset{-}{\gamma }$ is the mean value of *γ*. For a given set of parameter (*β*, $\overset{-}{\gamma }$, *θ*, *κ*, *B*, and *p*), we used equations (1–3) to generate expected SFSs for the three types of mutations. These SFSs were then combined into a single SFS to imitate ignoring gBGC. A combined SFS for neutral variants were generated in the same way (with parameters *θ*, *κ*, *B*, and *p*). As in the data analysis, the combined neutral SFS was fitted to a two-epoch model in DFE-*α* (in all cases the two-epoch model provided a significantly better fit than the constant-size model). Then, the combined selected SFS was used to estimate *β* and $\overset{-}{\gamma }$ conditional on the estimated demographic model, denoted by *β_ig-gBGC_* and ${\overset{-}{\gamma }}_{ig-gBGC}$, respectively. These were used to evaluate the integral in the following equations for obtaining expected values of *α* and *ω_a_*:

(8) $$\alpha =1-\frac{K_{4}}{K_{0}}\int \frac{\gamma }{1-e^{-\gamma }}f_{d}(\gamma )d\gamma$$

and

(9) $$\omega_{a}=\frac{K_{0}}{K_{4}}-\int \frac{\gamma }{1-e^{-\gamma }}f_{d}(\gamma )d\gamma$$

where *K*_0_ and *K*_4_ represent the expected divergence level at selected and neutral sites, and *f_d_*(*γ*) is the probability density function of the DFE for deleterious variants. To calculate *K*_0_ we evaluated equations (5–7) using the true values of *β*, $\overset{-}{\gamma }$, *B*, *κ*, and *p*. *K*_4_ was calculated using *B*, *κ*, and *p.* For both *K*_0_ and *K*_4_, *Tθ* was arbitrarily set to 1, as this term is cancelled when taking the ratio between *K*_0_ and *K*_4_.

To understand the effects of positive selection, we generated data using a second type of DFE, in which a fraction *x* of new mutations are beneficial with selection coefficient *γ_x_*, whereas the remaining 1 − *x* are deleterious with *γ* following a reflected gamma distribution. Because our interest is to understand the effects of ignoring gBGC, but not the existence of positively selected SNPs in the SFS, on the estimation of the DFE, we obtained *β_ig-gBGC_* and ${\overset{-}{\gamma }}_{ig-gBGC}$ by first excluding positively selected variants from the combined SFS (i.e., by removing contributions from the proportion *x* of sites where mutations are beneficial). As above, the combined neutral SFS was fitted to a two-epoch model in DFE-*α*, and true parameter values (including *x* and *γ_x_*) were used to calculate *K*_0_ and *K*_4_, whereas *β_ig-gBGC_* and ${\overset{-}{\gamma }}_{ig-gBGC}$ were used to calculate the integrals in equations (8) and (9).

Using equations (5–7) and true values of the parameters, we can calculate “true *α*” by obtaining the substitution rate of deleterious mutations (depending on 1—*x*, *β*, $\overset{-}{\gamma }$, *κ*, *B*, and *p*) and the substitution rate of beneficial mutations (depending on *x*, *γ_x_*, *κ*, *B*, and *p*). Thus, true *α* informs us what proportion of substitutions has beneficial effects on fitness (i.e., *γ *> 0). “True *ω_a_*” can be calculated in a similar way. When all sites are used, substitution patterns may be affected by both gBGC and natural selection (i.e., *B* ≠ 0, as opposed to *B* = 0 or when only WWSS sites are used). True *α* and true *ω_a_* provide a way to understand how the joint effects of these two processes affect the prevalence of adaptive substitutions.

It should be noted that we used the *expected* SFSs and expected divergence levels generated by the equations in the above analysis. This is justified because our interest is not the statistical property of the inference procedure, but the *expected* effects of ignoring gBGC on the estimation of *β*, $\overset{-}{\gamma }$, *α*, and *ω_a_*. The validity of this procedure can be seen by the fact that, when *B* = 0, the values of *β*, $\overset{-}{\gamma }$, *α*, and *ω_a_* produced by the above method are very close to the true values (table 1 and supplementary table S8, Supplementary Material online). The minor deviations were caused by numerical differences between our model, which generates data using diffusion equations (eqs. 1–7), and DFE-*α*, which uses a matrix approach (Keightley and Eyre-Walker 2007). This suggests that, when *B* ≠ 0, the *β_ig-gBGC_* and ${\overset{-}{\gamma }}_{ig-gBGC}$ values produced by the procedure should be those that best approximates the combined selected SFS.

**Table 1:** The Effects of Ignoring gBGC on the Estimation of *β* and $\overset{-}{\gamma }$, *α*, and *ω_a_*

B	True α		True ω_a_		DFE-α Results, All Sites, Ignoring gBGC			
	WWSS	All Sites	WWSS	All Sites	β	$\overset{-}{\gamma }$	α	ω_a_
Case 1. Parameters: *β* = 0.3, $\overset{-}{\gamma }$ = −200, *x* = 0								
0	0	0	0	0	0.303	−197.3	0.0108	0.0018
1	0	0	0	0	0.304	−196.4	0.0216	0.0036
3	0	0	0	0	0.295	−209.1	0.0590	0.0106
5	0	0	0	0	0.279	−231.5	0.0893	0.0175
10	0	0	0	0	0.244	−292.5	0.1306	0.0305
Case 2. Parameters: *β* = 0.3, $\overset{-}{\gamma }$ = −2000, *x* = 0.005, *γ_x_* = 3								
0	0.1588	0.1588	0.0158	0.0158	0.303	−1938.0	0.1685	0.0167
1	0.1588	0.1573	0.0158	0.0157	0.304	−1921.8	0.1756	0.0175
3	0.1588	0.1255	0.0158	0.0129	0.298	−2072.3	0.1816	0.0187
5	0.1588	0.0960	0.0158	0.0104	0.286	−2369.3	0.1865	0.0202
10	0.1588	0.0641	0.0158	0.0080	0.259	−3316.2	0.2065	0.0257
Case 3. Parameters: *β* = 0.2, $\overset{-}{\gamma }$ = −40, *x* = 0.03, *γ_x_* = 10								
0	0.4442	0.4442	0.3000	0.3000	0.2017	−40.0	0.4478	0.3025
1	0.4442	0.4479	0.3000	0.3058	0.2027	−40.0	0.4557	0.3111
3	0.4442	0.3882	0.3000	0.2502	0.1930	−42.9	0.4099	0.2642
5	0.4442	0.3065	0.3000	0.1843	0.1781	−47.5	0.3417	0.2055
10	0.4442	0.1801	0.3000	0.1027	0.1492	−57.8	0.2402	0.1370

Note.—The parameters values common to all three cases are *θ* = 0.01, *κ* = 2, *P* = 0.472, and *n* = 50. The number of neutral and selected sites are both 5 × 10^6^. True *α* and True *ω_a_* were calculated analytically using true parameter values (see Materials and Methods), whereas the *α* and *ω_a_* values derived from DFE-*α* were based on evaluating equations (8) and (9) using estimates of *β* and $\overset{-}{\gamma }$ obtained when gBGC was ignored (see Materials and Methods).

## Results

### Polymorphism Data Suggest Zebra Finches Have a Significantly Larger Effective Population Size than Great Tits

We performed whole-genome resequencing of ten wild European great tits, to a high depth of coverage for each individual (∼40–50×; supplementary table S1, Supplementary Material online). SNP calling and quality filtering were based on the GATK best practice guidelines (Van der Auwera et al. 2013); see Materials and Methods. We identified ∼10.4 million autosomal biallelic SNPs (supplementary table S2, Supplementary Material online). The autosomal diversity level calculated over all available variants was *π *= 0.0032. For zebra finch, we downloaded previously published whole-genome data (∼18–22× depth; supplementary table S1, Supplementary Material online) for ten wild zebra finches from mainland Australia (Singhal et al. 2015). Using the same SNP calling and quality filtering procedures, we identified ∼32.6 million biallelic SNPs and estimated an autosomal nucleotide diversity of *π *= 0.0086 (supplementary table S2, Supplementary Material online), which is very similar to the estimate of *π *= 0.0082 reported by Singhal et al. (2015).

**Fig. 1.**
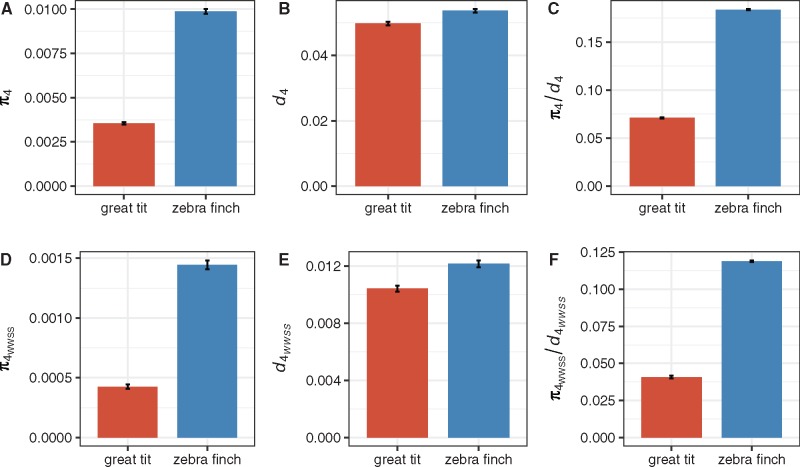
—Diversity (*π*) and divergence (*d*) at 4-fold sites in the great tit and zebra finch lineages. Panels (*A*–*C*) were based on all variants, and panels (*D*–*F*) were based on WWSS changes.

The expected level of neutral diversity in a population is determined by the product of the mutation rate per site per generation (*u*) and the effective population size (i.e., for diploid organisms: *E*(*π*)* = *4*N_e_u*). Using 4-fold degenerate sites as the neutral reference, nucleotide diversity at these sites, *π*_4_, was 0.0035 in great tits, but was 2.83 times larger at 0.0099 in zebra finches (bootstrapping *p* < 0.05; fig. 1*A*;supplementary table S3, Supplementary Material online). To control for the possibility that these two species may have very different mutation rates, we estimated divergence at 4-fold sites (*d*_4_) for both the great tit and zebra finch lineages by using the chicken genome as an outgroup. Given that base composition is not at equilibrium in many avian lineages (Nabholz et al. 2011; Mugal et al. 2013; Weber et al. 2014), a model that produces highly accurate results even in the presence of nonequilibrium base composition was employed (Matsumoto et al. 2015). As can been seen from figure 1*B* (see also supplementary table S5, Supplementary Material online), *d*_4_ is 1.1 times greater in the zebra finch lineage, indicating that the 2.83-fold difference in *π*_4_ cannot be attributed entirely to differences in the mutation rate. In fact, noting that *π*_4_/*d*_4_ is 0.070 and 0.186 for great tits and zebra finches, respectively, and appealing to the fact that *N_e_* is proportional to *π*_4_/*d*_4_, the observed *π*_4_/*d*_4_ ratios suggest that *N_e_* is 2.66-times higher zebra finches (bootstrapping *P* < 0.05; fig. 1*C*), consistent with the conclusion derived from *π*_4_ alone.

As described in the Introduction, gBGC, despite being a neutral process, can create a selection-like pattern favouring G/C nucleotides (referred to as the S (for “strong”) allele) over A/T nucleotides (referred to as the W (for “weak”) allele). This could have affected our analysis by distorting diversity patterns for both neutral and selected variants. Given the clear evidence supporting the existence of gBGC in birds (Webster et al. 2006; Nabholz et al. 2011; Mugal et al. 2013; Weber et al. 2014; Smeds, Mugal, et al. 2016), we repeated our analysis by using only W to W and S to S changes (denoted hereafter as WWSS changes), which are expected to be unaffected by gBGC (fig. 1*D*–*F*). As can be seen, the difference in *N_e_* becomes more pronounced between the two species—*N_e_* in zebra finches is 3.40 and 2.92 times higher based on *π*_4_ and *π*_4_/*d*_4_, respectively. These results suggest that controlling for gBGC may indeed be important for obtaining reliable results.

### Both Nonsynonymous Sites and UTRs Are under Selective Constraints and Purifying Selection Is More Effective in Zebra Finches

**Fig. 2.**
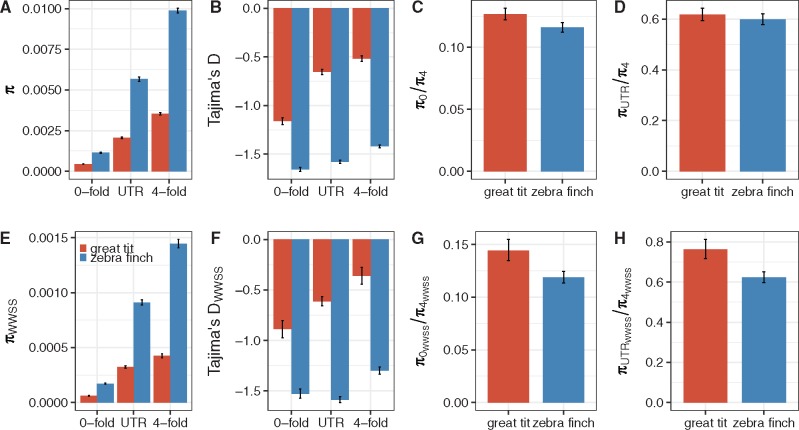
—Comparing diversity patterns across different types of sites and between the two species. Panels (*A*–*D*) were based on all variants, and panels (*E*–*H*) were based on WWSS changes.

Figure 2*A* shows that, in both species and based on all variants, *π*_0_ (nucleotide diversity at 0-fold degenerate sites) is significantly smaller than *π*_UTR_ (nucleotide diversity in UTRs; bootstrapping *P* < 0.05), which is in turn significantly smaller than *π*_4_ (bootstrapping *P* < 0.05). These observations suggest that both nonsynonymous sites and UTRs are under selective constraints, and that selection on nonsynonymous positions is stronger. This is further supported by the fact that the site-frequency spectra for both 0-fold and UTR sites harbored significantly more low-frequency variants than those for 4-fold sites (as suggested by the more negative Tajima’s *D*; bootstrapping *P* < 0.05; fig. 2*B*).

Comparing results based on all variants from the two species, *π*_0_/*π*_4_ is significantly lower in zebra finches than in great tits (bootstrapping *P* < 0.05; fig. 2*C*;supplementary table S3, Supplementary Material online), suggesting that, compared with the great tit genome, a higher proportion of new nonsynonymous mutations in the zebra finch genome are being strongly selected against and make little contribution to polymorphisms. This is consistent with purifying selection having higher efficacy in zebra finches, the species with a significantly higher *N_e_*. The trend is similar for UTRs, with a lower *π*_UTR_/*π*_4_ observed in zebra finches than in great tits (fig. 2*D*). However, the confidence intervals overlap considerably between the two species, and the *π*_UTR_/*π*_4_ ratios are not significantly different (bootstrapping *P* = 0.12). This may be a result of the complex interaction between recent demographic changes and differences in the shape of the DFE, which are not considered by *π*_UTR_/*π*_4_. Another possibility is the confounding effects of gBGC. As shown in figure 2*E*–*H*, the WWSS-based results are qualitatively very similar to those based on all variants, but the differences between species with respect to both *π*_0_/*π*_4_ and *π*_UTR_/*π*_4_ become more pronounced, with both statistics being significantly lower in the zebra finch (bootstrapping *P* < 0.05). We also found that this is not an artifact caused by the subsampling of sites for WWSS analyses (supplementary table S4, Supplementary Material online). These results again imply that ignoring gBGC could potentially mask important signals in the data.

The fact that Tajima’s *D* for 4-fold sites is significantly different from zero and negative in both species, especially in zebra finches, clearly indicates recent changes in population size (fig. 2*B* and *F*;supplementary table S3, Supplementary Material online). To further investigate the role of purifying selection in shaping polymorphism patterns, taking into account nonequilibrium dynamics, we estimated the DFE for both nonsynonymous and UTR sites using the method of Keightley and Eyre-Walker (2007), which assumes that the DFE follows a gamma distribution. As above we employed 4-fold sites as the neutral reference, and fitted the neutral SFS to a “two-epoch” model with a recent, single step change in population size. Consistent with the results based on Tajima’s *D*, there is clear evidence for a recent population expansion in both species and the extent of growth is more conspicuous in zebra finches (supplementary table S6, Supplementary Material online). Comparisons of the observed and expected SFS for the neutral data indicate that the demographic model provides a good fit to the data (supplementary fig. S1, Supplementary Material online).

**Fig. 3.**
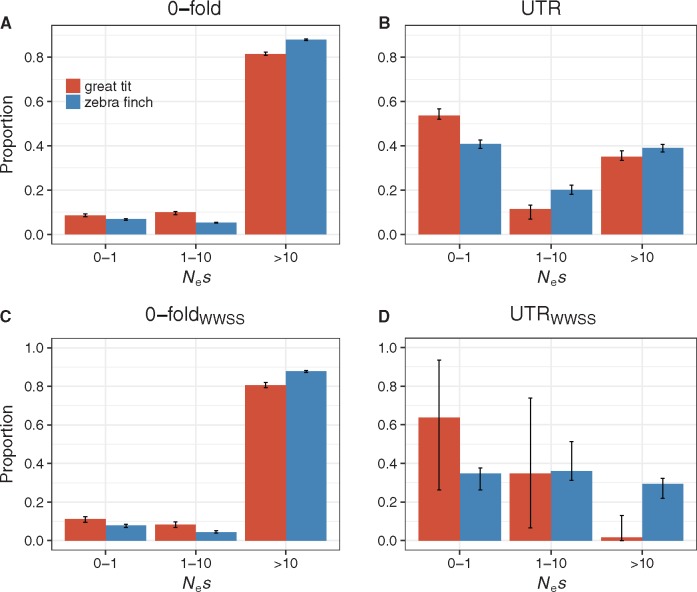
—Proportions of the DFE falling into different *N_e_s* ranges in the two species. Panels (*A*) and (*B*) were based on applying DFE-*α* to all changes, and panels (*C*) and (*D*) were based on WWSS changes.

For both nonsynonymous sites and UTRs, the maximum likelihood estimates of the shape parameter of the gamma distribution (β) are significantly <1, suggesting that the DFE is highly leptokurtic (supplementary table S6, Supplementary Material online). There is some evidence that the DFE for the UTRs is more leptokurtic than that for the nonsynonymous sites, especially in great tits, although the UTR estimates can be rather noisy (supplementary table S6, Supplementary Material online). Because the mean strength of purifying selection (i.e., mean *N_e_s*) is difficult to estimate reliably (Keightley and Eyre-Walker 2007), it cannot be used as a reliable indicator of the level of purifying selection. We therefore estimated the proportions of new mutations that are nearly neutral (*N_e_s* < 1), subject to intermediate level of selection (1 < *N_e_s* < 10), and strongly deleterious (*N_e_s* > 10). For the 0-fold sites, great tits have significantly more nearly neutral variants and variants under intermediate level of selection, regardless of whether all variants or WWSS variants were used (fig. 3*A* and *C*; bootstrapping *P* < 0.05), consistent with the results derived above using *π*_0_/*π*_4_ (fig. 2*C* and *G*). The estimates for UTRs also reveal clear evidence for significantly weaker purifying selection in great tits, as can be seen by the excess of nearly neutral variants and the rarity of strongly deleterious variants in this species relative to zebra finches (fig. 3*B* and *D*). However, the use of WWSS changes inevitably reduces the size of the data (about an 8-fold reduction in the number of SNPs) and the confidence intervals are therefore wider, especially for the UTRs (fig. 3*B* vs. 3*D*). This observation has implications for our analyses on genes grouped into different categories (discussed later).

### Positive Selection Is Widespread in Both Species and Is More Prevalent in Zebra Finches Even after Taking into Account the Confounding Effects of gBGC

**Fig. 4.**
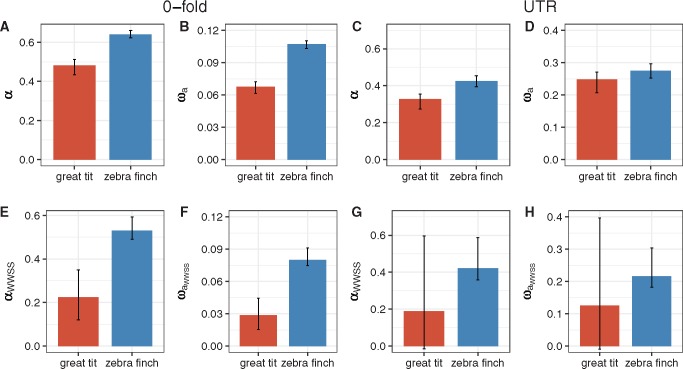
—Estimates of *α* and *ω_a_* for 0-fold and UTR sites in both species using either all variants or only WWSS changes. Results for 0-fold sites are in (*A*), (*B*), (*E*), and (*F*). Results for UTRs are in (*C*), (*D*), (*G*), and (*H*). (*A*–*D*) were obtained by analyzing all variants, whereas *E*–*H* were based on WWSS changes.

We investigated how common adaptive substitutions are at 0-fold and UTR sites in the two species, and whether the differences in *N_e_* between species would also translate into differences in the rate of adaptive evolution experienced by each species (Eyre-Walker and Keightley 2009). Using the DFE estimates obtained in the previous section and the lineage-specific estimates of *d*_0_ (divergence at 0-fold sites) and *d*_4_, we estimated the proportion of substitutions driven by positive selection, *α* (Eyre-Walker and Keightley 2009). Positive selection is widespread in both species. Based on all 0-fold and UTR variants, *α* = 48% and 33%, respectively, in great tits, and *α* = 64% and 43%, respectively, in zebra finches (fig. 4*A* and *C*; all these estimates are significantly >0; bootstrapping *P *< 0.05). Furthermore, *α* at both 0-fold and UTR sites is significantly higher in zebra finches than in great tits (fig. 4*A* and *C*; all variants; bootstrapping *P *< 0.05). To rule out the possibility that the larger α value in zebra finches is due to more effective purging of deleterious variants, rather than more rapid fixation of beneficial ones, we also calculated the relative rate of adaptive evolution *ω_a_*(Gossmann et al. 2010). Zebra finches have higher *ω_a_* for both 0-fold sites and UTRs (fig. 4*B* and *D*; all variants; bootstrapping *P* < 0.05 in both cases), supporting the theoretical prediction that adaptive substitutions occur at a higher rate in the species with a larger *N_e_*.

To examine what impact gBGC may have on estimates of *α* and *ω_a_*, we reestimated both parameters using WWSS variants only. This results in a reduction in the estimates of *α* and *ω_a_*in both species for both 0-fold and UTR sites (fig. 4*E*–*H*), suggesting that gBGC may lead to overestimation of the level of adaptive substitutions. As we observed for *π*_0_/*π*_4_ and *π*_UTR_/*π*_4_, the differences between the two species with respect to both *α* and *ω_a_* become more pronounced when WWSS sites were used (fig. 4*A*–*D* vs. 4*E*–*H*). The zebra finch lineage still has significantly higher *α* and *ω_a_* at 0-fold sites than the great tit lineage (bootstrapping *P* < 0.05 for both statistics; fig. 4*E* and *F*). For the UTRs, although the point estimates of both statistics are smaller in great tits (fig. 4*G* and *H*), the widths of the confidence intervals in this species have increased so much that they overlap zero, and neither statistic was found to be significantly different between the two species (bootstrapping *P* > 0.1). Given this large increase in statistical noise in the UTR estimates, and also the fact that UTRs were only available for a subset of the genes (see Materials and Methods), we focused on 0-fold sites only in the next section.

### Clear Evidence for More Effective Purifying Selection in Regions of High Recombination, but Mixed Evidence for Positive Selection

**Fig. 5.**
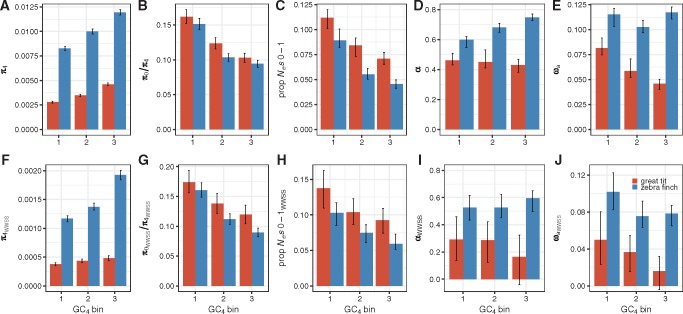
—Comparing polymorphism and divergence patterns across the three GC_4_ bins between great tits and zebra finches. The genes were grouped into three equal-sized bins based on their GC content at 4-fold degenerate sites (GC_4_), with bin 1, 2, 3 containing genes with low, intermediate, high GC_4_, respectively. The results presented in (*A*–*E*) were based on analyzing all changes, whereas those in (*F*–*J*) were based on WWSS changes only. (*C*) and (*H*) show the proportion of the DFE falling into the nearly neutral range (i.e., 0 ≤ *N_e_s* ≤ 1).

To examine the effects that recombination rate variation has on the efficacy of selection in each species’ genome, we grouped the genes into three bins (see Materials and Methods). We used a small number of bins mainly due to the limited number of WWSS variants available. In figure 5*A*–*E*, we present results derived from binning genes into three equal-sized groups according to their GC content at 4-fold sites (GC_4_). This is reasonable because GC_4_ is highly correlated with local recombination rates in these two species (Spearman’s *ρ* = 0.43, *P* < 2.2 × 10^−16^ and *ρ* = 0.37, *P* < 2.2 × 10^−16^ for great tit and zebra finch, respectively), so that the bin membership of a gene should be reflective of the long-term recombination environment it has been exposed to.

The conclusions reached the previous sections regarding between-species differences in diversity level (fig. 5*A* and *F*), the efficacy of purifying selection (fig. 5*B*, *C*, *G*, and *H*), and the prevalence of adaptive nonsynonymous substitutions (fig. 5*D*, *E*, *I*, and *J*) remain valid across the bins, suggesting that these results hold genome-wide and are not driven by a small handful of genes.

Regardless of whether all variants or WWSS changes were used, three trends were observed in both species (fig. 5): *π*_4_ increase with GC_4_ whereas both *π*_0_/*π*_4_ and the proportion of nearly neutral nonsynonymous variants in the DFE (i.e., those with *N_e_s* falling in [0, 1]) decrease with GC_4_. These patterns are all indicative of a higher efficacy of purifying selection in regions with more frequent recombination.

Contrary to the HRI theory’s prediction of higher rates of adaptive substitution in high-recombination regions, no consistent trends were observed for *α* and *ω_a_*. Based on all variants, *α* increases with GC_4_ in zebra finches (e.g., bin 3 has a significantly higher *α* than both bins 1 and 2; bootstrapping *P* < 0.05; fig. 5*D*), but we see the opposite in great tits, although the differences between bins are nonsignificant (fig. 5*D*). The *ω_a_* estimates suggest that the positive relationship between *α* and GC_4_ in zebra finches is probably mostly due to more efficient purging of deleterious mutations by purifying selection, as bins 1 and 3 have nearly identical *ω_a_* (fig. 5*E*). For great tits, *ω_a_* is in fact significantly lower in the high-recombination regions (bootstrapping *P* < 0.05; fig. 5*E*).

When *α* and *ω_a_* were estimated using WWSS variants, the positive relationship between *α* and GC_4_ in zebra finches is weakened, with no significant differences found between bins (fig. 5*I*), and *ω_a_* becomes significantly smaller in high-recombination regions (bin 1 vs. bin 3; bootstrapping *P* < 0.05; fig. 5*J*). A similar strengthening of the negative relationship between GC_4_ and *α* (or *ω_a_*) is also observed in great tits. This is mainly caused by a more pronounced drop in both *α* and *ω_a_*in high-recombination regions when only WWSS changes were used (e.g., in great tits, comparing fig. 5*D–I*, the reduction in *ω_a_*for bin 1 and bin 3 is 38.9% and 65.2%, respectively; in zebra finches, the reduction for the same two bins is 11.7% and 33.2%, respectively).

To check the robustness of our results, we repeated the above analyses with an additional binning strategy. This strategy is based on a measure of the density of putatively selected sites per centiMorgan (see Materials and Methods), which should control for the fact that there is a positive correlation between gene density and recombination in birds (Backstrom et al. 2010). The results derived from this second binning strategy are largely consistent with those reported above (supplementary fig. S2, Supplementary Material online). However, an interesting exception is observed in zebra finches where both *α* and *ω_a_* increase as the density of putatively selected sites decreases, both before and after gBGC was taken into account (supplementary fig. S2*I* and *J*, Supplementary Material online).

The results presented in figure 5 therefore convey two important messages. First, although the HRI theory correctly predicts the covariation between the efficacy of purifying selection and recombination, it fails to explain the variation of the prevalence of adaptive substitutions across different genomic regions. Second, gBGC is an important confounding factor for the study of selection, and there is evidence that the bias caused by gBGC is greatest in high-recombination regions where its effects are expected to be stronger.

### Theoretical Analysis of a gBGC Model

To investigate the effects of gBGC further, we developed a model that takes into account GC content (47.2% at 0-fold sites in both species), mutational bias towards A/T nucleotides (e.g., Smeds, Qvarnström, et al. 2016), the DFE, and the GC-favoring effects of gBGC (see Materials and Methods). The model was used to generate SFS and divergence data for both neutral and selected sites, under various strengths of gBGC, as measured by *B*. These data were then analyzed by DFE-α by first fitting a two-epoch demographic model to the neutral variants, and the DFE was then estimated using the selected variants conditional on the estimated demographic model. All these analyses were done without regard to the presence of gBGC (i.e., analyzing all variants rather than just WWSS changes). Several cases based on parameter values realistic for the two birds of interest are displayed in table 1 (see supplementary table S8, Supplementary Material online for more results).

It is evident that the effects of gBGC cannot be totally controlled for by fitting a demographic model to the neutral variants. The estimates of *β* and $\overset{-}{\gamma }$ in table 1 become smaller as gBGC becomes stronger. Biases in *β* and $\overset{-}{\gamma }$ caused by ignoring gBGC affect the estimation of *α* and *ω_a_*. In fact, ignoring gBGC may result in either false detection of adaptive evolution (Case 1 in table 1), or overestimation of both *α* and *ω_a_* relative to their true values when positive selection is present (Cases 2 and 3 in table 1). The difference between the estimates of *α* and *ω_a_* obtained from ignoring gBGC (last two columns in table 1) and those based on WWSS variants (second and fourth columns in table 1) is more complicated. When positive selection is infrequent (Cases 1 and 2), estimates derived from ignoring gBGC can be higher than those based on WWSS variants, but the reverse may be true when positive selection is more prevalent (Case 3). There is some tentative evidence for these behaviors in our data. Adaptive substitutions are less frequent in great tits, and the drop in *α* is more visible: 48% based on all variants and 22% based on WWSS variants. In zebra finches, the corresponding estimates are 64% and 53%. However, we have not attempted to reproduce these quantities using the model, due to the lack of detailed information about some important parameters (i.e., the mutation matrix and *B*).

Given that there is evidence for a positive correlation between recombination rate and the strength of gBGC in a number of species (Weber et al. 2014; Glémin et al. 2015; Singhal et al. 2015; Wallberg et al. 2015), we also examined how gBGC may affect the study of HRI within a genome by changing the value of *B* while holding all other parameters constant. We used true parameter values to analytically calculate *true α*, the proportion of substitutions that has beneficial effects on fitness (i.e., *γ *> 0; see Materials and Methods). We notice that the *true α* based on all variants tends to decrease with increasing *B* (Cases 2 and 3). This is partly caused by gBGC hindering the fixation of advantageous S→W mutations, and partly caused by an increased fixation rate of slightly deleterious W→S mutations driven by gBGC. However, the *true α* based on WWSS variants is invariant with respect to *B*. Interestingly, when positive selection is nonexistent (Case 1) or infrequent (Case 2), ignoring gBGC can create a false positive relationship between *α* (or *ω_a_*) and *B* (or recombination; see the last two columns). In contrast, when there are frequent adaptive substitutions (Case 3), the relationship between *α* (or *ω_a_*) and *B* (or recombination) can be negative. These results suggest that gBGC can complicate studies of HRI. Interestingly, in all three cases, relative to the true value based on all variants (Columns 3 and 5), the extent of overestimation of *α* and *ω_a_* (the last two columns) caused by ignoring gBGC is an increasing function of *B* (e.g., in Case 3, *α* is overestimated by 1.7% and 33.3% for *B* = 1 and 10, respectively).

Comparing the HRI results in figure 5 to the theoretical results, we note some qualitative similarities: 1) relative to the results based on all variants, the WWSS-based estimates of *α* (and *ω_a_*) are reduced across all bins in both species, and the extent of reduction tends to be more significant in high GC_4_ bins (cf. Cases 1 and 2 in table 1, Column 2 vs. Column 8); 2) in great tits, the negative relationship between GC_4_ and the estimates of *α* (and *ω_a_*) based on all variants can potentially be caused by gBGC being more effective in slowing down fixation of beneficial mutations in high-recombination regions (cf. Cases 2 and 3 in table 1, Column 2). Since the model predicts that results based on WWSS variants should not be affected by gBGC, the variation in *α* and *ω_a_* estimates presented in figure 5*I* and *J* (see also supplementary fig. S2*I* and *J*, Supplementary Material online) suggest that there might indeed be some difference in the efficacy of positive selection across the genome, although the direction of the difference is often inconsistent with predictions of the HRI theory.

## Discussion

In this study, we used whole-genome polymorphism data sets from two passerine birds (great tit and zebra finch) to quantify the level of purifying and positive selection. In addition to coding regions, we also obtained, to our knowledge, the first estimates of *α* and *ω_a_* for UTRs in birds. Our results show that the vast majority (>80%) of new nonsynonymous mutations and a significant proportion (>30%) of new UTR mutations are subject to strong purifying selection in both species (*N_e_s* > 10; fig. 3). This finding agrees with an earlier study by Künstner et al. (2011a) which reported that 3′UTRs have evolved under evolutionary constraint in birds, and with a recent study of the collared flycatcher that reported reduced diversity in UTR regions relative to 4-fold sites and other noncoding regions (Dutoit et al. 2017). In zebra finches, after controlling for gBGC, the proportions of 0-fold and UTR substitutions driven by positive selection were estimated to be 53% and 42%, respectively (supplementary table S7, Supplementary Material online); the corresponding estimates are lower in great tits at 22% and 19% respectively. These results show that both purifying and positive selection are widespread in these birds, and that the intensity of selection on UTRs is comparable to those reported in other organisms (e.g., in *Mus musculus* 25% of UTRs are under strong purifying selection, and the estimates in *Capsella grandiflora* are 12% and 21% for 5′ and 3′ UTRs; for *α* in UTRs, it is ∼60% in *D. melanogaster*, 19% in *M. musculus*, and 39% and 28% for 5′ and 3′ UTRs in *C. grandiflora*) (Andolfatto 2005; Halligan et al. 2013; Williamson et al. 2014). We have also studied possible determinants of the efficacy of selection, both between species and within the genome. Although we have obtained clear evidence that selection is more effective in zebra finch, the species with a larger *N_e_*, the situation is nonetheless more complex within the genomes of each species. Although the efficacy of purifying selection increases with predictors of higher local *N_e_* in both species, the relationship between *ω_a_* and the predictors of local *N_e_* is often negative or nonsignificant, especially after gBGC has been taken into account (fig. 5 and supplementary fig. S2, Supplementary Material online). The implications of our findings are discussed below.

### The Importance of Controlling for gBGC When Studying Selection

There is a growing body of literature, showing that gBGC plays an important role in the evolution of many organisms, including microbes (e.g., Lesecque et al. 2013), plants (e.g., Glémin et al. 2014), and animals (e.g., Glémin et al. 2015). Estimates of the strength of gBGC, as measured by *B*, often fall in the range 0 ≤ *B* ≤ 1 (Spencer et al. 2006; Muyle et al. 2011; De Maio et al. 2013), but there is clear evidence that *B* varies across the genome and can be well above ten in recombination/gBGC hotspots (Glémin et al. 2015). Previous studies have shown that gBGC can cause fixation of slightly deleterious variants (Galtier et al. 2009) and lead to erroneous detection of positive selection using *d_N_*/*d_S_*-based methods (Ratnakumar et al. 2010).

An interesting observation is that, when WWSS changes were used, the difference between the two species with respect to *π*_0_/*π*_4_ (fig. 2) and *α* (or *ω_a_*; fig. 4) become more pronounced, lending stronger support to the predicted effects of *N_e_* on the efficacy of selection. A similar observation was made in a study attempting to test the predicted correlation between *d_N_*/*d_S_* and *N_e_* in placental mammals (Lartillot 2013), in which the observed relationship became consistent with the model prediction only after *d_N_*/*d_S_* was calculated on WWSS changes. These examples illustrate that not controlling for gBGC can also obscure genuine signals in the data (see also Romiguier and Roux 2017).

Here we show, both theoretically and empirically, that gBGC can bias estimates of the DFE, *α* and *ω_a_* (fig. 4 and table 1). Interestingly, the effects of ignoring gBGC cannot be controlled for by fitting a two-epoch demographic model to neutral variants in DFE-*α* (table 1). This is different from other confounding factors such as linked selection for which the demography fitting approach has been shown to be effective (e.g., Eyre-Walker and Keightley 2009; Messer and Petrov 2013). Studying HRI within a genome can also be complicated by gBGC in that, when gBGC is ignored, *α* (or *ω_a_*) and *B* can be either positively (Cases 1 and 2 in table 1) or negatively correlated (Case 3 in table 1), even though the strength of positive selection and the rate at which beneficial mutations arise are both constant across the genome. Thus, if *B* and recombination is correlated, as has been shown in a number of species (Weber et al. 2014; Glémin et al. 2015; Singhal et al. 2015; Wallberg et al. 2015), ignoring gBGC could potentially lead to misleading conclusions regarding how recombination modulates the efficacy of selection. Finally, our analysis of the model suggests that the effect of gBGC on quantifying selection is complex, and is dependent on parameters that are often poorly known (e.g., the mutation matrix). Thus, exploring to what extent gBGC can explain the quantitative differences we observed between the all-variant-based and WWSS-based results is an important avenue for future research.

### The Effects of Other Potential Confounding Factors

When using WWSS variants to control for the effects of gBGC, we have also removed C → T and G → A transitions arising at methylated CpG sites, which occur at high rates due to rapid deamination. In humans, there is some evidence that, in protein-coding regions, transitions occurring at CpG sites are under stronger selective constraints than those occurring in non-CpG contexts, but no such difference was detected for transversions occurring inside and outside CpG contexts (Schmidt et al. 2008). If this is also true in birds, some of the differences we observed between the results based on all sites and those based on WWSS sites could be caused by CpG hypermutability. A difficulty is that C → T and G → A transitions at CpG sites are also S → W mutations, which are disfavored by gBGC. This makes it nontrivial to separate the effects of purifying selection from those of gBGC at these sites. Another possible complication is that both methylation levels and the strength of gBGC are positively correlated with recombination rates in humans and birds (Glémin et al. 2015; Mugal et al. 2015; Singhal et al. 2015). Thus, we might expect these two forces to covary. Given that both CpG methylation and gBGC are common phenomena, there is a pressing need for more studies to identify the relative importance of these two forces in shaping genome evolution, and clarify whether there are systematic differences between CpG and non-CpG variants, as well as between WWSS variants and other variants, with respect to their effects on fitness. However, it should be noted that, if 0-fold C → T and G → A transitions at CpG sites are also under stronger constraints in birds, their inclusion should make estimates of *α* based on all variants smaller. But our estimates based on all variants are consistently higher than those based on WWSS. This suggests that CpG effects may not be a major contributor to the observation, which seems reasonable in light of the lower level of CpG methylation in birds (the frequency of methylated CpG sites in chicken is 0.0037, compared with 0.0062 in humans; Mugal et al. 2015).

There is evidence that 4-fold sites may be under selective constraints in birds (Künstner et al. 2011b). An alternative is to use ancestral repeats (ARs) as neutral reference. Unfortunately, repetitive regions pose a particularly difficult challenge for variant calling using short-read data (Li 2014), and are routinely removed from these analyses (e.g., Singhal et al. 2015). To assess the impact of using 4-fold sites on our results, we calculated nucleotide diversity using SNPs in ARs, denoted by *π*_AR_. In zebra finches, *π*_4_/*π*_AR_ = 0.86; in great tits, *π*_4_/*π*_AR_ = 0.78. Assuming that the issue of reduced SNP calling reliability in ARs did not exist, and that the reduction in diversity level at 4-fold sites is due entirely to selective constraints, then 4-fold sites in great tits appear to under strong selection than in zebra finches. It is known that selective constraints at 4-fold sites lead to overestimation of *α* and the extent of overestimation increases with the level of constraint (Matsumoto et al. 2016). Thus, the observation that 4-fold sites may be under strong constraints in great tits should make our suggestion that the great tit lineage has a significantly smaller *α* conservative.

### Prevalence of Adaptive Substitutions in Birds

In supplementary table S9, Supplementary Material online, we present estimates of *α* for nonsynonymous changes obtained in three previous studies of various avian species. Comparing these to our results (22% in great tits and 52% in zebra finches, both based on WWSS changes) is not straightforward because, first, none of the previous studies controlled for gBGC, and second, whereas we used lineage-specific divergence in our analysis, the previous studies used the total divergence from the focal species to the outgroup. Bearing these in mind, we set out to explore other possible sources of the difference. In chickens (using the zebra finch as an outgroup), *α* has been estimated at ∼20% (Axelsson and Ellegren 2009) and ∼0% (Downing et al. 2009), considerably lower than our estimate for the zebra finch. As acknowledged by Axelsson and Ellegren (2009), their estimate may be downwardly biased for two reasons. First, the chicken population has probably experienced dramatic demographic changes (e.g., a domestication bottleneck) and intense artificial selection, which are known to cause an increase in the proportion of segregating slightly deleterious mutations (Charlesworth and Charlesworth 2010). To address this Axelsson and Ellegren (2009) removed low-frequency alleles from the polymorphism data. However, this procedure may still result in an underestimation of *α* (Charlesworth and Eyre-Walker 2008; Messer and Petrov 2013). Second, only genes expressed in the brain were used in the analysis, which have been shown to be under stronger constraint than genes expressed in other tissues (Axelsson et al. 2008). On the other hand, the very low estimates of adaptive evolution obtained by Downing et al. (2009) is likely due to the presence of slightly deleterious mutations in the chicken polymorphism data, and the fact that their effects were not controlled for in the estimation of *α*.

More recently, Galtier (2016) reported very high estimates of *α* and *ω_a_*for nonsynonymous changes in three species of birds (≥86% for *α* and ≥29% for *ω_a_*), with one of them, the blue tit, also being a passerine (supplementary table S9, Supplementary Material online). It is unlikely that this is due to the overrepresentation of highly expressed genes in the transcriptome-based approach employed because genes with high levels of expression tend to be more conserved (Pál et al. 2006). As none of the three species have higher diversity level than the zebra finch (supplementary table S9, Supplementary Material online), a difference in *N_e_* may not be the reason either; although, as discussed below, variation in *N_e_* between species may not be a very reliable predictor of the rate of adaptive substitution. The effects of other methodological differences (e.g., the effects of undetected paralogs on the transcriptome data [Gayral et al. 2013; Lopez-Maestre et al. 2016]) are hard to assess. One way to resolve the discrepancy is to obtain whole-genome resequencing data from these species and reestimate these two parameters after appropriately controlling for gBGC.

### Determinants of the Efficacy of Selection between Species

Because the contribution of a new mutation to both polymorphism and divergence is dependent on the composite parameter *γ* = 4*N_e_s* (Charlesworth and Charlesworth 2010), the efficacy of selection is affected by both *N_e_* and the distribution of fitness effects (DFE). Assuming that the DFE is similar, a larger *N_e_* should lead to a smaller proportion of segregating nearly neutral variants and a higher rate of adaptive substitution. These have been observed between the two avian species studied here (figs. 2–4) and in a number of between-species comparisons (Jensen and Bachtrog 2011; Strasburg et al. 2011; Gossmann et al. 2012; Phifer-Rixey et al. 2012; Wang et al. 2016). In agreement with the fact that most new mutations that have an effect on fitness are deleterious (Eyre-Walker and Keightley 2007), both *π_N_*/*π_S_* and *d_N_*/*d_S_* have been found to be negatively correlated with *N_e_* across species (Lartillot 2013; Figuet et al. 2016; Galtier 2016; Chen et al. 2017).

There are, however, notable exceptions (e.g., Bachtrog 2008; Andolfatto et al. 2011). For instance, *Drosophila miranda* has a 5-fold smaller *N_e_* than *D. melanogaster*, and yet the two species were found to have similar *π_N_*/*π_S_* and *α* (Bachtrog 2008). Several studies of birds have also reported a lack correlation between *N_e_* and *d_N_*/*d_S_* (Nabholz et al. 2013; Weber et al. 2014; Figuet et al. 2016). On the other hand, in a study involving the transcriptomes from 44 species pairs, Galtier (2016) found that, although *α* is positively correlated with *N_e_*, there is no evidence that *ω_a_* and *N_e_* are correlated. This suggests that there is no increase in the rate of adaptive substitution with *N_e_*, and that the positive relationship between *α* and *N_e_* is probably driven by more effective purging of deleterious variants in species with larger *N_e_*.

There are several possible explanations for the apparent discrepancy between theory and data discussed above. For example, Bachtrog (2008) suggested that *α* may have been overestimated for *D. miranda* because it was estimated on the total divergence between *D. miranda* and *D. pseudoobscura*, and it is known that *D. pseudoobscura* has a larger *N_e_* and a high rate of adaptive substitution (Jensen and Bachtrog 2011). It is also possible that the DFE is rather different between species (Eyre-Walker and Keightley 2007). For example, species with smaller *N_e_* may be, on average, further away from their fitness optimum, and are thus more likely to acquire strongly beneficial mutations (Bachtrog 2008; Galtier 2016). Additionally, if adaptive evolution is not mutation-limited, such that beneficial variants frequently interfere with one another (and with linked deleterious mutations), the dependency of the rate of adaptive substitution on *N_e_* can be substantially weakened (reviewed by Lanfear et al. 2014). Finally, simulations have provided evidence that, in a fluctuating environment, the relationship between the rate of adaptive evolution and *N_e_* may plateau once *N_e_* is above ten thousand or so (Lourenço et al. 2013). Interestingly, *d_N_*/*d_S_* and *π_N_*/*π_S_* continue to decline with increasing *N_e_* (Lourenço et al. 2013), suggesting that these two statistics may be relatively robust indicators of the efficacy of purifying selection. This is probably because deleterious mutations typically dominate the DFE, and thus the dynamics of the two statistics are less sensitive to details of the shape of the DFE. In contrast, *α* and *ω_a_* may depend more sensitively on the frequency and fitness effects of beneficial mutations.

In light of the above, the reported lack of correlation between *d_N_/d_S_* and *N_e_* in birds at both mitochondrial genes (Nabholz et al. 2013) and nuclear genes (Weber et al. 2014; Figuet et al. 2016) is surprising. However, a recent study by Botero-Castro et al. (2017) has shown that the inclusion of genes with high GC-content, previously excluded due to annotation and assembly issues, results in a significant correlation between *d_N_/d_S_* and proxies of *N_e_* in birds. In our data set, *d_N_*/*d_S_* is significantly higher in the zebra finch lineage, which has a larger *N_e_* (supplementary table S5, Supplementary Material online). However, this may be in part due to the much higher rate of adaptive substitution along this lineage (fig. 4). Overall, our results are consistent with the theory and points to selection being more effective in zebra finches (figs. 2–4). It is possible that our use of the DFE-*α* approach has allowed us to more accurately tease apart the relative contribution of positive and negative selection to molecular evolution. However, more studies with whole-genome polymorphism data from more avian species are necessary before a more definite answer can be formulated.

### Determinants of the Efficacy of Selection within a Genome

Within a genome, the efficacy of selection is also predicted by the Hill-Robertson interference (HRI) theory to increase with local *N_e_*. However, empirical studies have unearthed extreme disparities among species (Cutter and Payseur 2013). In some species such as *Drosophila* the efficacy of both positive and purifying selection clearly becomes higher in regions with more frequent recombination (Campos et al. 2014; Castellano et al. 2016), whereas in other species no such relationship can be found (e.g., Bullaughey et al. 2008; Slotte et al. 2011; Flowers et al. 2012). Here we have found evidence that the efficacy of purifying selection is higher in regions predicted to have larger *N_e_*, but observed no clear relationship between *ω_a_* and local *N_e_* (fig. 5 and supplementary fig. S2, Supplementary Material online). The inconsistency between the two types of selection may be due to the different sensitivity of *π*_0_/*π*_4_ and *ω_a_* to details of the DFE, as we speculated above. There are also other reasons why testing the HRI theory is particularly difficult. First, accurately predicting local *N_e_* is challenging. In most previous studies, the recombination rate is used as a proxy for local *N_e_*. However, under background selection, for instance, local *N_e_* is determined by exp(-*U*/*M*), where *U* is the deleterious mutation rate and *M* is the map length (Charlesworth 2012). Thus, if there is a strong positive relationship between recombination rate and the density of putatively selected sites, local *N_e_* may not increase with recombination, as has been observed in rice (Flowers et al. 2012). We addressed this issue by binning genes according to the *M*/*C* ratio (supplementary fig. S2, Supplementary Material online), where *C* is the number of protein-coding sites. However, the reliability of this approximation depends critically on the quality of the genome annotation, which is hard to assess. Second, in contrast to the >2-fold difference in *π*_4_ between the two species (fig. 1), the maximum difference in *π*_4_ between different bins is <1.5-fold (fig. 5). The smaller difference may mean that these between-bin comparisons are more susceptible to statistical noise.

It is also instructive to compare our results to previous studies of HRI in birds. Gossmann et al. (2014) aligned CDS fragments assembled from a great tit transcriptome data set to both the chicken and zebra finch reference genomes. By examining *d_N_*/*d_S_* (calculated on all variants) across regions with different recombination rates, they concluded that the efficacy of both positive and purifying selection is higher in regions of high recombination. Their conclusion regarding the efficacy of positive selection does not necessarily disagree with our finding here. First, the “site test” Gossmann et al. (2014) used is known to be highly conservative and probably only detects recurrent fixation of strongly beneficial alleles (<0.5% of the genes analyzed by Gossmann et al. (2014) were deemed statistically significant). Thus, they may not make a significant contribution to the DFE and the results reported here. Second, the “site test” was carried out on the total divergence between great tit and zebra finch. Thus, the weak evidence of a positive relationship between *ω_a_* and predictors of *N_e_* in the zebra finch lineage (fig. 5 and supplementary fig. S2, Supplementary Material online) may have contributed to their results. A recent study, by Bolívar et al. (2016), on the flycatcher genome, found that *θ*_0_/*θ*_4_ (when calculated on WW and SS variants) was not correlated with the recombination rate, and that the covariation between *d*_0_/*d*_4_ (calculated on all variants) and recombination is probably driven by gBGC, rather than varying intensity of purifying selection. The difference between the flycatcher and the two birds studied here is interesting, especially when considering that the level of divergence between these three species is rather similar (*d_S_* ranging between ten and 15% [Künstner et al. 2010; Gossmann et al. 2014]) and that there have been relatively few intrachromosome inversions since the species split (Kawakami et al. 2014; van Oers et al. 2014). However, while Botero-Castro et al. (2017) reported a strong positive correlation between *d_S_* and GC content in 44 species of birds, a relationship that is not predicted by the HRI theory, they also found a weak, but significant, negative correlation between *d_N_* and GC content, consistent with the HRI theory. The reason for these differences in the bird studies is unclear and warrants further investigation.

## Conclusion

Overall, it is evident that our understanding of what determines variation in the efficacy of selection between species and between different genomic regions is far from complete. Answering this important question requires not only the continual generation of high-quality data (reference genome and its annotation, polymorphism data, high-resolution genetic map, etc.) but also the development of new models to help us make better use of the data and understand the dependency of patterns of divergence and polymorphism on essential evolutionary parameters.

## Supplementary Material

Supplementary data are available at *Genome Biology and Evolution* online.

## Supplementary Material

Supplementary Data

Supplementary Data
