# Supplementary material for: Determinants of the Efficacy of Natural Selection on Coding and Noncoding Variability in Two Passerine Species
Source: Genome Biol Evol. 2017 Oct 17;9(11):2987–3007. doi: 10.1093/gbe/evx213 (PMC5714183; doi:10.1093/gbe/evx213)
Supplement: Supplementary Data [file evx213_suppfigandtables.docx]

**Supplementary Figures**

**Fig S1. Comparing the observed 4-fold site SFS and the SFS predicted by the best-fit demographic model.**

**
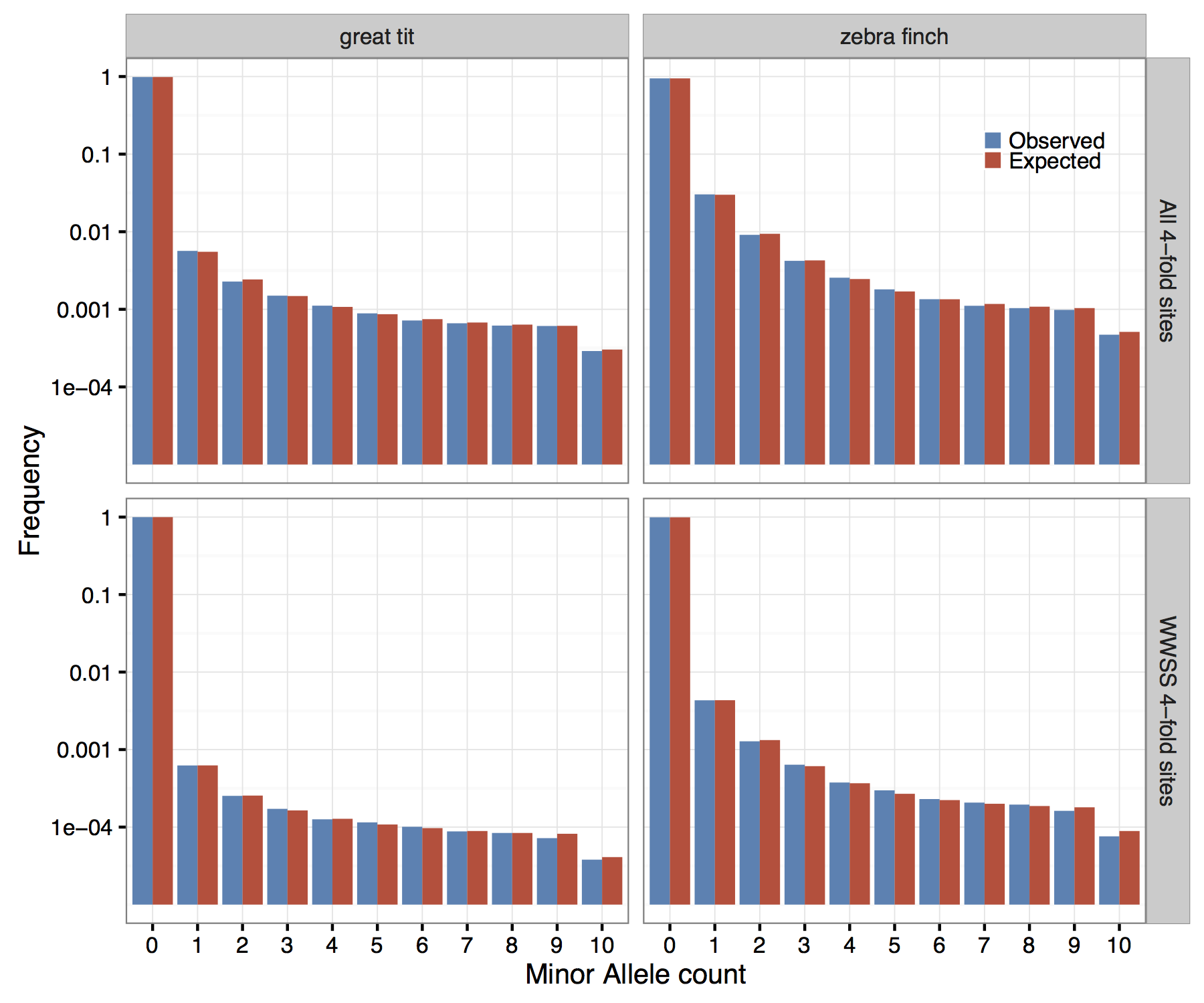
**

**Fig S2. Comparing polymorphism and divergence patterns across the three *M*/*C* bins between great tits and zebra finches.** The genes were grouped into three equal-sized bins based on their *M*/*C*, where *M* is the map length of the window centred around the midpoint of the gene and *C* is the number of coding sites in the window (see Methods). The results presented in (A) – (E) were based on analysing all changes, whereas those in (F) – (J) were based on WWSS changes only. (C) and (H) show the proportion of the DFE falling into the nearly neutral range (i.e., 0 ≤ *N_e_s* ≤ 1).

**Table S1. Sampling location and mean depth of coverage for samples of great tit (*Parus major*) and zebra finch (*Taeniopygia guttata*) used in this study.**

| **Sample id.** | ***Speccies*** | **Location** | **Mean depth**  **of coverage** |
| --- | --- | --- | --- |
| 1280 | *Parus major* | Mariola, Spain | 41.0 |
| 1485 | *Parus major* | Austria | 44.0 |
| 15 | *Parus major* | Pilis Mountains, Hungary | 47.0 |
| 167 | *Parus major* | Gotland, Sweden | 43.1 |
| 249 | *Parus major* | Loch Lomond, Scotland | 45.1 |
| 318 | *Parus major* | Oulu, Finland | 49.6 |
| 61 | *Parus major* | Velky Kosr, Czech Republic | 42.8 |
| 917 | *Parus major* | La Rouvière, France | 40.2 |
| 943 | *Parus major* | Pirio/Muro, Corsica, France | 47.7 |
| TR43666 | *Parus major* | Oxford, Great Britain | 44.0 |
| 26462 | *Taeniopygia guttata* | Fowlers Gap, New South Wales, Australia | 21.5 |
| 26733 | *Taeniopygia guttata* | Fowlers Gap, New South Wales, Australia | 21.5 |
| 26781 | *Taeniopygia guttata* | Fowlers Gap, New South Wales, Australia | 23.9 |
| 26795 | *Taeniopygia guttata* | Fowlers Gap, New South Wales, Australia | 21.4 |
| 26820 | *Taeniopygia guttata* | Fowlers Gap, New South Wales, Australia | 19.2 |
| 28078 | *Taeniopygia guttata* | Fowlers Gap, New South Wales, Australia | 22.2 |
| 28313 | *Taeniopygia guttata* | Fowlers Gap, New South Wales, Australia | 24.9 |
| 28353 | *Taeniopygia guttata* | Fowlers Gap, New South Wales, Australia | 21.2 |
| 28456 | *Taeniopygia guttata* | Fowlers Gap, New South Wales, Australia | 23.5 |
| 28481 | *Taeniopygia guttata* | Fowlers Gap, New South Wales, Australia | 17.7 |

**Table S2.** **Summary of the numbers of called sites (Sites) and biallelic autosomal SNPs (S) after VQSR and quality filtering in the population samples from the great tit and zebra finch.**

| **Species** | **Sites** | **S** | **𝜃_W_** | **𝜋** | **Tajima’s D** |
| --- | --- | --- | --- | --- | --- |
| great tit | 849382181 | 10433723 | 0.0035 | 0.0032 | -0.3785 |
| zebra finch | 696319113 | 32557756 | 0.0132 | 0.0086 | -1.4619 |

**Table S3. Summary of polymorphisms at all 0-fold and 4-fold sites and at WWSS sites**. S, the number of segregating sites (subscript 0: 0-fold degenerate sites; subscript 4: 4-fold degenerate sites); ***𝜋***, nucleotide diversity per site; **𝜃**, Watterson's theta per site. “Sites” specifies whether all-sites (‘All’) or only WWSS sites were used. Values in parentheses show 95% confidence intervals.

| **Species** | great tit | zebra finch | great tit | zebra finch |
| --- | --- | --- | --- | --- |
| **Sites** | All | All | WWSS | WWSS |
| **Genes** | 8095 | 7799 | 8095 | 7799 |
| **S_0_** | 20849 | 52906 | 2616 | 7553 |
| **S_4_** | 31419 | 95325 | 3620 | 13388 |
| **π_0_** | 0.000449 (0.000433, 0.000466) | 0.001145 (0.001108, 0.001183) | 6.1e-05 (5.8e-05, 6.5e-05) | 0.000172 (0.000165, 0.000179) |
| **π_4_** | 0.003541 (0.003471, 0.003612) | 0.009873 (0.009725, 0.010024) | 0.000425 (0.000407, 0.000444) | 0.001446 (0.001409, 0.001483) |
| **π_0_ / π_4_** | 0.126792(0.122209, 0.131598) | 0.115983(0.112319, 0.119774) | 0.144241 (0.134491, 0.154473) | 0.118765 (0.113403, 0.124279) |
| **θ_0_** | 0.00062 (0.000601, 0.00064) | 0.001891 (0.001834, 0.001949) | 7.8e-05 (7.4e-05, 8.2e-05) | 0.00027 (0.00026, 0.00028) |
| **θ_4_** | 0.004041 (0.00397, 0.004112) | 0.014919 (0.014698, 0.015144) | 0.000466 (0.000448, 0.000483) | 0.002095 (0.002048, 0.002143) |
| **θ_0_ / θ_4_** | 0.153561 (0.148683, 0.158551) | 0.126761 (0.122995, 0.130565) | 0.167232 (0.157799, 0.177245) | 0.128852 (0.123695, 0.134156) |
| **Tajima’s D_0_** | -1.16014 (-1.19481, -1.1263) | -1.65595 (-1.6769, -1.63509) | -0.88797 (-0.97334, -0.80354) | -1.52778 (-1.57325, -1.48148) |
| **Tajima’s D_4_** | -0.51887 (-0.54755, -0.49067) | -1.41978 (-1.43471, -1.40452) | -0.36105 (-0.44426, -0.27783) | -1.30125 (-1.3361, -1.26543) |

**Table S4. Mean π_0_ / π_4_ and π_UTR_ / π_4_ ratios calculated on the 10,000 subsampled datasets.** The subsamples were generated by randomly sampling (without replacement) from the full dataset the same numbers of 0-fold and 4-fold SNPs as in the WWSS dataset. The p-values in parentheses show whether the π_0_ / π_4_ and π_UTR_ / π_4_ are significantly different from the observed WWSS values.

|  | **great tit** | **zebra finch** |
| --- | --- | --- |
| **π_0_ / π_4_** | 0.138 (p < 0.01) | 0.118 (p > 0.05) |
| **π_UTR_ / π_4_** | 0.788 (p < 0.01) | 0.683 (p < 0.001) |

**Table S5. Estimates of substitution rates at 0-fold (d_0_) and 4-folds sites (d_4_) obtained with BASEML, and estimates of nonsynonymous (*d_N_*) and synonymous (*d_S_*) substitution rates obtained using CODEML for the set of autosomal orthologs in each species.** Values in parentheses show 95% confidence intervals.

|  | **great tit** | **zebra finch** |
| --- | --- | --- |
| **genes** | 8095 | 7799 |
| ***d*_0_** | 0.00699 (0.00679, 0.00720) | 0.00899 (0.00877, 0.00917) |
| ***d*_4_** | 0.04984 (0.04921, 0.05038) | 0.05377 (0.05312, 0.05430) |
| ***d*_0_ /*d*_4_** | 0.13870 (0.13799, 0.14294) | 0.16565 (0.16514, 0.16894) |
| ***d*_N_** | 0.00763 (0.00740, 0.00789) | 0.00977 (0.00954, 0.01000) |
| ***d*_S_** | 0.05203 (0.05146, 0.05246) | 0.05882 (0.05816, 0.05949) |
| ***d*_N_ /*d*_S_** | 0.14661 (0.14289, 0.15062) | 0.16617 (0.16217, 0.16927) |

**Table S6. Estimates of demographic parameters of the two-epoch model fitted to the SFS for 4-fold sites and parameters of the DFE for deleterious 0-fold and UTR sites.** Values in parentheses are the 95% confidence intervals. Population size before the step change is given by *N_1_* and after by *N_2_*. The time of the population size change is given by *t_2_.* The parameters of the DFE are: *β*, the shape parameter of the gamma distribution and *N_e_*$\bar{s}$, the product of the mean homozygous effect of a deleterious mutation and the effective population size.

| **Species** | **Selected sites^1^** | ***N*_2_ / *N*_1_**  **(95% C.I.)** | ***t*_2_ / *N_2_***  **(95% C.I.)** | ***N_e_***$\bar{s}$  **(95% C.I.)** | ***β***  **(95% C.I.)** |
| --- | --- | --- | --- | --- | --- |
| great tit | All 0-fold | 2.31  (2.31, 2.79) | 0.31  (0.17, 0.35) | 701  (568, 1427) | 0.34  (0.29, 0.35) |
| zebra finch | All 0-fold | 10  (10, 10) | 0.22  (0.21, 0.23) | 17802  (11292, 27963) | 0.25  (0.24, 0.27) |
| great tit | All UTR | 2.31  (2.31, 2.79) | 0.31  (0.17, 0.35) | 241  (119, 8798) | 0.08  (0.05, 0.10) |
| zebra finch | All UTR | 10  (10, 10) | 0.22  (0.21, 0.23) | 45  (32, 63) | 0.18  (0.16, 0.20) |
| great tit | WWSS 0-fold | 2.79  (1.91, 10) | 0.08  (0.01, 0.26) | 3306  (1083, 16100) | 0.24  (0.19, 0.29) |
| zebra finch | WWSS 0-fold | 8.79  (7.99, 8.79) | 0.18  (0.17, 0.22) | 163371  (23714, 9.6 x 10^5^) | 0.19  (0.16, 0.24) |
| great tit | WWSS UTR | 2.79  (1.91, 10) | 0.08  (0.01, 0.26) | 1  (0.85, 5) | 0.36  (0.11, 99.9) |
| zebra finch | WWSS UTR | 8.79  (7.99, 8.79) | 0.18  (0.17, 0.22) | 11  (6, 13) | 0.34  (0.28, 0.57) |

**^1.^**Selected sites column specifies whether all-sites (‘All’) or only WWSS sites were used as the selected sites in the DFE-alpha analysis.

**Table S7. Estimates of the proportion of adaptive substitutions (α) and rate of adaptive evolution relative to rate of neutral divergence (ω_a_) from all 0-fold sites, WWSS 0-fold sites, all UTR sites and WWSS UTR sites.**

| **Species** | **Selected sites^1^** | *α* **(95% C.I.)** | *ω_a_* **(95% C.I.)** |
| --- | --- | --- | --- |
| great tit | All 0-fold | 0.48 (0.43, 0.51) | 0.068 (0.061, 0.072) |
| zebra finch | All 0-fold | 0.64 (0.62, 0.66) | 0.107 (0.103, 0.110) |
| great tit | All UTR | 0.33 (0.27, 0.35) | 0.25 (0.21, 0.27) |
| zebra finch | All UTR | 0.43 (0.39, 0.45) | 0.28 (0.25, 0.29) |
| great tit | WWSS 0-fold | 0.22 (0.12, 0.35) | 0.029 (0.015, 0.044) |
| zebra finch | WWSS 0-fold | 0.53 (0.49, 0.59) | 0.080 (0.075, 0.091) |
| great tit | WWSS UTR | 0.19 (-0.01, 0.60) | 0.13 (-0.01,0.39) |
| zebra finch | WWSS UTR | 0.42 (0.36, 0.59) | 0.22 (0.18, 0.30) |

**^1.^**Selected sites column specifies whether all-sites (‘All’) or only WWSS sites were used as the selected sites in the DFE-alpha analysis.

**Table S8. The effects of ignoring gBGC on the estimation of** *β* and $\bar{}$**, *α*, and** *ω_a_***.** The parameters values common to all three cases are *θ* = 0.01, *κ* = 2, *p* = 0.472, and *n* = 50. The number of neutral and selected sites are both 5 × 10^6^.

| *B* |  | True *α* | |  | True *ω_a_* | |  | DFE-*α* results, all sites, ignoring gBGC | | | |
| --- | --- | --- | --- | --- | --- | --- | --- | --- | --- | --- | --- |
|  |  | WWSS | All sites |  | WWSS | All sites |  | *β* | $\bar{}$ | *α* | *ω_a_* |
| Parameters: *β* = 0.3, $\bar{}$ = -2000, *x* = 0 | | | | | | | | | | | |
| 0 |  | 0 | 0 |  | 0 | 0 |  | 0.303 | -1938.0 | 0.0115 | 0.0010 |
| 1 |  | 0 | 0 |  | 0 | 0 |  | 0.304 | -1921.8 | 0.0217 | 0.0018 |
| 3 |  | 0 | 0 |  | 0 | 0 |  | 0.298 | -2072.3 | 0.0641 | 0.0058 |
| 5 |  | 0 | 0 |  | 0 | 0 |  | 0.286 | -2369.3 | 0.1002 | 0.0099 |
| 10 |  | 0 | 0 |  | 0 | 0 |  | 0.259 | -3316.2 | 0.1521 | 0.0178 |
| Parameters: *β* = 0.3, $\bar{}$ = -60, *x* = 0 | | | | | | | | | | | |
| 0 |  | 0 | 0 |  | 0 | 0 |  | 0.302 | -60.02 | -0.0147 | -0.0035 |
| 1 |  | 0 | 0 |  | 0 | 0 |  | 0.304 | -59.90 | 0.0438 | 0.0106 |
| 3 |  | 0 | 0 |  | 0 | 0 |  | 0.290 | -63.78 | 0.2167 | 0.0581 |
| 5 |  | 0 | 0 |  | 0 | 0 |  | 0.269 | -70.13 | 0.2368 | 0.0698 |
| 10 |  | 0 | 0 |  | 0 | 0 |  | 0.225 | -85.82 | 0.1097 | 0.0387 |
| Parameters: *β* = 0.3, $\bar{}$ = -200, *x* = 0.005, *γ_x_* = 5 | | | | | | | | | | | |
| 0 |  | 0.1599 | 0.1599 |  | 0.0316 | 0.0316 |  | 0.303 | -197.3 | 0.1599 | 0.0316 |
| 1 |  | 0.1599 | 0.1584 |  | 0.0316 | 0.0314 |  | 0.304 | -196.4 | 0.1765 | 0.0350 |
| 3 |  | 0.1599 | 0.1264 |  | 0.0316 | 0.0258 |  | 0.295 | -209.1 | 0.1780 | 0.0364 |
| 5 |  | 0.1599 | 0.0967 |  | 0.0316 | 0.0208 |  | 0.279 | -231.5 | 0.1774 | 0.0382 |
| 10 |  | 0.1599 | 0.0646 |  | 0.0316 | 0.0160 |  | 0.244 | -292.5 | 0.1868 | 0.0461 |
| Parameters: *β* = 0.3, $\bar{}$ = -5, *x* = 0.05, *γ_x_* = 10 | | | | | | | | | | | |
| 0 |  | 0.5161 | 0.5161 |  | 0.5000 | 0.5000 |  | 0.290 | -5.26 | 0.5161 | 0.5000 |
| 1 |  | 0.5161 | 0.5194 |  | 0.5000 | 0.5096 |  | 0.293 | -5.29 | 0.5244 | 0.5145 |
| 3 |  | 0.5161 | 0.4539 |  | 0.5000 | 0.4170 |  | 0.250 | -5.72 | 0.4657 | 0.4278 |
| 5 |  | 0.5161 | 0.3623 |  | 0.5000 | 0.3072 |  | 0.198 | -6.32 | 0.3756 | 0.3185 |
| 10 |  | 0.5161 | 0.2149 |  | 0.5000 | 0.1712 |  | 0.126 | -7.42 | 0.2370 | 0.1887 |

**Table S9. Estimates of the proportion of non-synonomous substitutions fixed by positive selection *(𝛼*) and rate of adaptive evolution relative to rate of neutral divergence (*𝜔_a_*) in different species of birds.**

| **Species** | **Outgroup^1.^** | ***π*_s_ ^2.^** | ***α***  **(95% CI)** | ***ω_a_***  **(95% CI)^3.^** | **Study** |
| --- | --- | --- | --- | --- | --- |
| *Parus caeruleus*  (blue tit) | *Parus major*  (great tit) | 0.00534 | 0.93  (0.87, 1.00) | 0.47  (0.43, 0.50) | (Galtier 2016) |
| *Aptenodytes patagonicus*  (king penguin) | *Aptenodytes forsteri*  (emperor penguin) | 0.00263 | 0.93  (0.83, 1.04) | 0.45  (0.40, 0.50) | (Galtier 2016) |
| *Eudyptes moseleyi*  (northern rockhopper penguin) | *Pygoscelis papua*  (gentoo penguin) | 0.00296 | 0.86  (0.80, 0.92) | 0.29  (0.27, 0.31) | (Galtier 2016) |
| *Gallus gallus*  (chicken) | *Taeniopygia guttata*  (zebra finch) | 0.00841 | 0.20 | NA | (Axelsson and Ellegren 2009) |
| *Gallus gallus*  (chicken) | *Taeniopygia guttata*  (zebra finch) |  | -0.09 | NA | (Downing et al. 2009) |

^1.^ In all the previous studies divergence data was obtained by pairwise comparison with an outgroup. Therefore, the estimates of divergence are not lineage specific.

^2.^ Estimates for species in Galtier (2016) obtained from Romiguier et al. (2014). Estimates for *Gallus gallus* are the mean of π_s_ values in Tables S2 in (Wong et al. 2004) .

^3.^ The *𝜔_a_* was not calculated in all studies.
