# Supplementary material for: Determinants of the Efficacy of Natural Selection on Coding and Noncoding Variability in Two Passerine Species
Source: Genome Biol Evol. 2017 Oct 17;9(11):2987–3007. doi: 10.1093/gbe/evx213 (PMC5714183; doi:10.1093/gbe/evx213)
Supplement: Supplementary Data [file evx213_supp.docx]

**The Great Tit HapMap Consortium**

Frank Adriaensen, Eduardo Belda, Andrey Bushuev, Mariusz Cichon, Anne Charmantier, Niels Dingemanse, Blandine Doligez, Tapio Eeva, Kjell Einar Erikstad, Slava Fedorov, Martin A. M. Groenen, Michaela Hau, Sabine Hille, Camilla Hinde, Bart Kempenaers, Anvar Kerimov, Milos Krist, Veronika Laine, Raivo Mand, Erik Matthysen, Reudi Nager, Claudia Norte, Markku Orell, Heinz Richner, Ben C. Sheldon, Tore Slagsvold, Vallo Tilgar, Joost Tinbergen, Janos Torok, Barbara Tschirren, Kees Van Oers, Koen J. F. Verhoeven, Marcel Visser, & Tera Yuta

**Affiliations:**

1. Edward Grey Institute, Department of Zoology, University of Oxford, Oxford OX1 3PS, UK
   - Ben C. Sheldon

##### Department of Animal Ecology, Netherlands Institute of Ecology (NIOO-KNAW), PO Box 50, Wageningen 6700 AB, The Netherlands

##### Veronika Laine

##### Kees Van Oers

##### Marcel Visser

### Animal Breeding and Genomics Centre, Wageningen University, P.O. Box 338, 6700AH Wageningen, The Netherlands

##### Marcel Visser

##### Martien A. M. Groenen

##### Department of Terrestrial Ecology, Netherlands Institute of Ecology (NIOO-KNAW), P.O. Box 50, 6700AB Wageningen, The Netherland

##### Kone J. F Verhoeven

##### Faculty of Biology, Lomonosov Moscow State University, Moscow 119234, Russia.

- - Andrey Bushuev
  - Anvar Kerimov

1. Inst. of Environmental Sciences, Jagiellonian University, Gronostajowa 7, 30-387 Kraków, Poland.
   - Mariusz Cichon
2. CEFE-CNRS, UMR 5175, 1919, route de Mende, F34293 Montpellier Cedex 5, France.
   - Anne Charmantier
3. Max Planck Institute for Ornithology, Department of Behavioural Ecology & Evolutionary Genetics, Eberhard-Gwinner-Straße, House 5, 82319 Seewiesen (Starnberg), Germany.
   - Niels Dingemanse
   - Michaela Hau
   - Bart Kempenaers
4. UMR CNRS 5558—LBBE, Biométrie et Biologie Évolutive, UCB Lyon 1 - Bât. Grégor Mendel, 43 bd du 11 novembre 1918, 69622 VILLEURBANNE cedex, France.
   - Blandine Doligez
5. Section of Ecology, Department of Biology, University of Turku, Turku 20014, Finland.
   - Tapio Eeva
6. Norwegian Institute for Nature Research, FRAM-High North Research Centre for Climate and the Environment, 9296 Tromsø, Norway.
   - Kjell Einar Erikstad
7. Department of Vertebrate Zoology, Moscow State University, Moscow 119899 Russia.
   - Slava Fedorov
8. Institute of Wildlife Biology and Game Management, University of Natural Resources and Life Science, A-1180 Vienna, Austria.
   - Sabine Hille
9. Behavioural Ecology Group, Department of Animal Sciences, Wageningen University, Wageningen 6708 PB, The Netherlands.
   - Camilla Hinde
10. Department of Zoology and Laboratory of Ornithology, Faculty of Science, Palacký University, Olomouc 77147, Czech Republic.
    - Milos Krist
11. Department of Zoology, Institute of Ecology and Earth Sciences, University of Tartu, Vanemuise 46, Tartu 51014, Estonia.
    - Raivo Mand
    - & Vallo Tilgar
12. Institute of Biodiversity, Animal Health and Comparative Medicine, University of Glasgow, Glasgow G12 8QQ, UK.
    - Reudi Nager
13. Department of Life Sciences, Institute of Marine Research IMAR/CMA, University of Coimbra, Coimbra, Portugal.
    - Claudia Norte
14. Department of Biology, University of Oulu, P.O. Box 3000, 90014 Oulu, Finland.
    - Markku Orell
15. Evolutionary Ecology Lab, Institute of Ecology and Evolution, University of Bern, Bern 3012, Switzerland.
    - Heinz Richner
16. Centre for Ecological and Evolutionary Synthesis (CEES), Department of Biosciences, University of Oslo, P.O. Box 1066, Blindern, 0316 Oslo, Norway.
    - Tore Slagsvold
17. Centre for Ecological and Evolutionary Studies (CEES), Univ. of Groningen, PO Box 11103, NL-9700 CC Groningen, The Netherlands.
    - Joost Tinbergen
18. Behavioural Ecology Group, Department of Systematic Zoology and Ecology, Eötvös Loránd University, Budapest H-1117, Hungary.
    - Janos Torok
19. Institute of Evolutionary Biology and Environmental Studies, University of Zurich, Winterthurerstrasse 190, CH-8057 Zurich, Switzerland.
    - Barbara Tschirren
20. Graduate School of Environmental Science, Hokkaido University, N10 W5 Sapporo, Hokkaido 060-0810, Japan.
    - Tera Yuta
